# A mixed relaxed clock model

**DOI:** 10.1098/rstb.2015.0132

**Published:** 2016-07-19

**Authors:** Nicolas Lartillot, Matthew J. Phillips, Fredrik Ronquist

**Affiliations:** 1 Laboratoire de Biométrie et Biologie Evolutive, UMR CNRS 5558, Université Claude Bernard Lyon 1, F-69622 Villeurbanne Cedex, France; 2 School of Earth, Environmental and Biological Sciences, Queensland University of Technology, Brisbane, Australia; 3 Department of Bioinformatics and Genetics, Swedish Museum of Natural History, PO Box 50007, 104 05 Stockholm, Sweden

**Keywords:** molecular dating, relaxed molecular clock, Bayesian inference, Monte Carlo

## Abstract

Over recent years, several alternative relaxed clock models have been proposed in the context of Bayesian dating. These models fall in two distinct categories: uncorrelated and autocorrelated across branches. The choice between these two classes of relaxed clocks is still an open question. More fundamentally, the true process of rate variation may have both long-term trends and short-term fluctuations, suggesting that more sophisticated clock models unfolding over multiple time scales should ultimately be developed. Here, a mixed relaxed clock model is introduced, which can be mechanistically interpreted as a rate variation process undergoing short-term fluctuations on the top of Brownian long-term trends. Statistically, this mixed clock represents an alternative solution to the problem of choosing between autocorrelated and uncorrelated relaxed clocks, by proposing instead to combine their respective merits. Fitting this model on a dataset of 105 placental mammals, using both node-dating and tip-dating approaches, suggests that the two pure clocks, Brownian and white noise, are rejected in favour of a mixed model with approximately equal contributions for its uncorrelated and autocorrelated components. The tip-dating analysis is particularly sensitive to the choice of the relaxed clock model. In this context, the classical pure Brownian relaxed clock appears to be overly rigid, leading to biases in divergence time estimation. By contrast, the use of a mixed clock leads to more recent and more reasonable estimates for the crown ages of placental orders and superorders. Altogether, the mixed clock introduced here represents a first step towards empirically more adequate models of the patterns of rate variation across phylogenetic trees.

This article is part of the themed issue ‘Dating species divergences using rocks and clocks’.

## 1. Introduction

It is now universally recognized that the substitution rate undergoes substantial variation through time. Accordingly, much effort has been devoted to the problem of accounting for rate heterogeneity across branches in molecular dating studies. To that effect, Bayesian dating methods rely on explicit models of rate variation. Over the years, a large series of alternative models have been developed, which can be divided into two main groups. On the one side, uncorrelated models assume that substitution rates are independent across successive branches of the phylogeny [1–4]. On the other side, autocorrelated models explicitly account for long-range dependencies in the evolution of the substitution rate over the tree. The most well-known example of an autocorrelated model is the lognormal (or Brownian) relaxed clock, which makes the assumption that the logarithm of the substitution rate evolves according to a Brownian motion [3,5,6]. Other autocorrelated clock models have been proposed, among which the compound Poisson process [7], the Ornstein–Uhlenbeck (OU) process [8], the Cox–Ingersoll–Ross (CIR) process [2] or the random local clocks [9].

Which class of relaxed clock models, autocorrelated or not, provides a more adequate choice for empirical analyses is still an open question. Model comparison, using Bayes factors or posterior predictive goodness-of-fit tests, has led to contradictory results, sometimes supporting autocorrelated clocks, sometimes instead uncorrelated models [1,2,10,11]. In some cases, different conclusions might have been the result of differing taxon sampling depth [2]. Alternatively, the choice between alternative relaxed clock models might depend on the evolutionary scale under consideration. For instance, a viral phylogeny may be well described by an uncorrelated clock. By contrast, on a macro-evolutionary scale, long-term changes in the substitution rate are expected. In mammals, for instance, there is a strong negative correlation between the rate of evolution and body size, probably owing, at least in nuclear genomes, to a generation-time effect [12–15]. Given the long-term trends in body-size evolution across mammals, where entire orders are globally characterized by either a small or a large body mass, this correlation implies that rate variation in mammals is fundamentally dominated by long-range autocorrelation. Similar broad-scale correlations between substitution rate and life-history traits are observed in other groups of vertebrates, as well as in invertebrates and in plants [16–19], which at first sight argues in favour of the use of autocorrelated relaxed clocks, rather than uncorrelated clocks, for molecular dating analyses on a large evolutionary scale. On the other hand, violations of the Brownian assumption have been pointed out [14], suggesting that current autocorrelated models may potentially result in biases in divergence time estimation.

Molecular comparative analyses, by formalizing the problem of estimating the correlation between rate and body mass in terms of multivariate Brownian processes [20], also emphasize that the rate of substitution is fundamentally a quantitative trait [21]. As such, it should ideally be modelled as a continuous-time process, undergoing continuous changes and/or discrete jumps at any time along the tree. This is in contrast with some of the clock models currently implemented, which are directly formulated in terms of the distribution of rates across branches. Such branch-wise models may be statistically and computationally convenient. However, they typically lack a clear mechanistic interpretation, in terms of macro-evolutionary processes.

Among currently existing uncorrelated clocks, the only model having a well-defined continuous-time representation is the white noise process [2]. This model can be seen as an extreme case of the CIR process (for the gamma white noise) or the OU process (for the lognormal white noise), in which the relaxation time is infinitely short. In practice, the white noise process is expected to represent a reasonable statistical description of a stochastic model of rate variation through time that would have a well-defined stationary distribution and a decorrelation time shorter than the typical length of the branches of the phylogenetic tree. On the other end of the parameter range, for low relaxation rates (long relaxation times), the OU process reduces to the classical lognormal or log-Brownian autocorrelated clock. Similarly, the CIR with low relaxation rate reduces to a model where the substitution rate evolves according to a squared Brownian process. Thanks to these parametric relationships between models, the problem of explicitly choosing between an autocorrelated and uncorrelated rate processes could be simply avoided by using by default a CIR or an OU clock model. Under well-designed parametrizations and priors, the data would then automatically tune the relaxed clock to the empirically most relevant regime.

The automatic model selection just suggested still implies a choice between mutually exclusive relaxed clock models, each entirely focused on one specific time scale. Yet, the true process of rate variation may consist of a superposition of long- and short-term aspects, in which case neither the pure Brownian nor the pure white noise process, nor even any intermediate OU or CIR regime, would represent an empirically adequate model. Incidental observations suggest that variation in the rate of molecular evolution may indeed unfold simultaneously over multiple time scales. For instance, in the context of rate/trait comparative analyses in mammals, it was found that taking averages over families increases the strength of the correlations between substitution rate and traits [22]. This suggests the existence of short-term fluctuations increasing the dispersion of the signal at the fine-grained species level, which are averaged out at the family level. In addition, there are known molecular evolutionary phenomena, in particular recombination-induced GC-biased-gene conversion [23], which can induce transient bursts of substitutions, locally along the genome [24,25]. Such phenomena could result in substantial fluctuations of the short-term rate of substitution, which may not be properly accommodated by current autocorrelated clocks.

To account for such complex patterns of rate variation, in this article, a mixed clock model is introduced. According to this model, rate variation has both a long-term Brownian behaviour (presumably driven by long-term changes in life-history strategies) and short-term variations, the latter being captured by a white noise process. By tuning the relative variance of its two components, the mixed clock interpolates between the uncorrelated and correlated relaxed clocks, containing them as particular cases. Applying this mixed clock model to an empirical dataset leads to a simple measure of how the total variation in substitution rate across the tree is partitioned into the Brownian and the white noise components, which then yields potentially useful insight about how rate variation unfolds over different time scales.

As with any other relaxed clock model, the mixed clock introduced here can be used in the context of both tip-dating and node-dating approaches. Here, node-dating refers to the formalization of fossil calibrations in terms of prior distributions directly imposed on a subset of nodes of the phylogeny [26–28]. Tip-dating in contrast, refers to an alternative molecular dating paradigm, which explicitly accounts for the fact that fossils and extant taxa are part of the same macro-evolutionary process [29–32]. This macro-evolutionary process is typically formalized in terms of a birth–death process with serial species sampling through time [31–33]. In the context of tip-dating, calibration of divergence times by fossils is implicit, being mediated by the prior on divergence times induced by the serial birth–death process [32] and, in the case where discrete morphological characters are used, the relaxed morphological clock [29,30]. For those theoretical reasons, tip-dating is currently emerging as an attractive alternative to node-dating.

On the other hand, in part because it offers less opportunity for locally controlling the impact of specific fossil calibrations, tip-dating may show—or perhaps more fundamentally reveal—a strong sensitivity of the dating analysis to the choice of the relaxed clock model and of the prior on divergence times. In this context, complex interactions between the priors on times and on rates could make the interpretation of formal model comparison or selection less straightforward. For instance, model selection may just favour the relaxed clock model that happens to be most compatible with the prior on divergence times. If this prior does not represent an empirically adequate model of the true species diversification and sampling processes, the relaxed clock model, in spite of having a higher fit, may nevertheless substantially mis-specify the patterns of rate variation.

Therefore, in the following analysis, the mixed clock was applied in parallel to the same set of 105 extant placental mammals, using both node-dating and tip-dating approaches. The node-dating analysis was provided with a rich set of fossil calibrations, so as to ensure that divergence times estimated in this context are not too sensitive to variations of the underlying relaxed molecular clock. This node-dating analysis was then used as a benchmark for testing alternative relaxed clock models, to then better understand the behaviour of those alternative relaxed clocks in the tip-dating context. Importantly, the analysis that follows is not meant as a benchmark of tip-dating versus node-dating. The two methods are not provided with comparable fossil information, and thus, no meaningful conclusion can be drawn, as to their relative merits, from the results now presented.

## 2. Results

### (a) Simulations

A series of simulations were conducted, followed by re-analysis under various relaxed clock models. These simulations were designed to assess to what extent the mixed clock is able, under the regimes effectively encountered in the empirical analyses, to correctly identify situations where rate variation has been produced by one of the two pure relaxed clocks, especially by the pure Brownian clock. Since the two components of the mixed clocks are additive on the logarithmic scale, the variance in the logarithm of the branch-specific substitution rates can be partitioned into the two contributions, thus providing a simple quantitative measure of the relative importance of the two components of the mixed clock. In order to ensure that the conclusions of these simulations are relevant for the empirical situations considered here, simulations were calibrated against parameter estimates obtained from the empirical analyses. For each setting, 10 replicates were simulated (see ‘Methods’).

In the context of node-dating, simulations were conducted under each of the three relaxed clocks (pure Brownian, pure white noise and mixed), using parameters, including divergence times, randomly drawn from the posterior distribution. In this series of analyses, pure clocks were always correctly recovered, in the sense that the mixed clock essentially reduces to whichever pure clock was used for simulating the data (electronic supplementary material, table S1). Thus, if data are simulated under the pure Brownian clock and then analysed under the mixed clock, at most 3% of the total rate variation is incorrectly attributed to the white noise component. Conversely, if data are simulated under the white noise clock and then analysed under the mixed clock, the fraction of total rate variation allocated to the Brownian component reaches at most 4%. Finally, when simulations are conducted under a mixed clock using the proportions defined by the random draw from the posterior distribution, the mixed clock infers qualitatively correct proportions, within an error of 9% on average, although for some replicates the error can be as large as 22%.

In the context of tip-dating, several analyses were conducted, which differed in the way divergence times were chosen for the simulation step. In a first series, divergence times were drawn from the prior distribution (the serial birth–death prior with diversified sampling) and were combined with parameter values randomly drawn from the posterior distribution (under the mixed clock). Analyses were then conducted under the matching prior. In this context, pure clocks are again correctly recovered, with at most 12% of the total rate variation attributed to the white noise component under a pure Brownian clock and at most 2% attributed to the Brownian component under a pure white noise clock (electronic supplementary material, table S2).

A second series of simulations were conducted using divergence times randomly drawn from a Yule prior without diversified sampling. These simulated data were then re-analysed under a serial birth–death prior not accounting for diversified sampling. Under this mis-specified prior settings, the proportion of variance incorrectly attributed to the white noise component for data simulated under a pure Brownian clock is substantial (24% on average) and can reach up to 58% (electronic supplementary material, table S3). This illustrates how biases in the estimation of divergence times induced by an incorrectly specified prior on divergence times can result in biases in the estimation of rates, which in turn leads to the inference of an incorrect relaxed clock regime. Inference in the other direction is more robust: at most 2% of the total variation is attributed to the Brownian component if the data are simulated under a pure white noise clock.

A third series of analyses were conducted under divergence times drawn from the posterior distribution (as in the case of node-dating above). In this context, up to 45% of the total rate variation (20% on average) is incorrectly attributed to the white noise component (electronic supplementary material, table S3). Given that the tree estimated by the Brownian clock departs from the expectation under the serial birth–death prior, this can be seen as a particular case of mis-specification of the prior on divergence times.

Altogether, what can be concluded from these simulations is that clock model selection is reliable in the context of the richly informed node-dating settings considered in this study. By contrast, in the context of tip-dating, the interactions between the relaxed clock model and the prior on divergence times are more difficult to control and can sometimes result in inadequate model selection. These observations justify the use of an independent assessment of alternative relaxed clock models in the context of a node-dating analysis with rich fossil calibration, before transposing the conclusions to the tip-dating case.

### (b) Empirical analyses using the node-dating approach

A standard node-dating approach was first conducted, using a modified version of the dataset of Meredith *et al.* [34], a large set of hard fossil constraints and under two alternative relaxed clock models, the autocorrelated Brownian (lognormal) clock [5] and the uncorrelated white noise clock [2].

The estimates under the two relaxed clock models are globally similar (tables 1 and 2; electronic supplementary material, figures S1,S2). At the level of orders and superorders, the main differences concern the age of Eulipotyphla, Euarchontoglires, Afrotheria and Placentalia which are all older by approximately 10 Myr under the white noise clock than under the Brownian clock (tables 1 and 2). As for the credible intervals, they are about twice as large under the white noise clock (average size of 14.3 Myr across all nodes), compared with the Brownian model (average size of 7.5 Myr).

**Table 1.** Estimates (posterior median and 95% credibility intervals) of divergence times in several placental orders under various model settings.

	age (million years)						
	Primates	Rodentia	Chiroptera	Carnivora	Perissodactyla	Cetartiodactyla	Eulipotyphla
node Brownian	63 (61,64)	66 (64,67)	61 (60,61)	59 (54,63)	59 (56,61)	63 (61,65)	72 (67,77)
node white noise	65 (57,76)	70 (65,76)	60 (58,61)	59 (51,65)	58 (56,61)	63 (59,66)	81 (71,90)
node mixed	67 (61,75)	67 (64,71)	60 (58,61)	61 (54,66)	58 (56,61)	64 (61,66)	79 (70,87)
tip Brownian	92 (85,101)	85 (79,93)	62 (54,72)	60 (55,66)	59 (49,67)	67 (62,72)	84 (75,92)
tip white noise	63 (52,75)	68 (59,77)	58 (51,67)	52 (44,60)	43 (37,52)	58 (53,64)	73 (61,85)
tip mixed	67 (58,78)	60 (54,70)	56 (49,65)	54 (47,62)	45 (38,55)	61 (55,67)	69 (59,80)
tip prior	49 (37,66)	55 (47,65)	56 (44,70)	53 (42,67)	49 (38,59)	57 (52,63)	54 (31,85)

**Table 2.** Estimates (posterior median and 95% credibility intervals) of crown divergence times for the four placental superorders and for Placentalia under various model settings.

	age (million years)				
	Euarchontoglires	Laurasiatheria	Xenarthra	Afrotheria	Placentalia
node Brownian	77 (73,81)	77 (73,81)	65 (59,71)	66 (62,73)	88 (83,94)
node white noise	87 (78,97)	83 (75,92)	66 (59,71)	74 (63,87)	100 (91,112)
node mixed	86 (78,94)	85 (78,92)	66 (59,71)	73 (64,84)	100 (92,108)
tip Brownian	104 (96,114)	95 (88,103)	74 (64,87)	76 (67,89)	120 (108,135)
tip white noise	84 (75,95)	79 (71,87)	56 (43,70)	66 (55,78)	95 (85,107)
tip mixed	80 (72,90)	77 (71,85)	60 (49,74)	65 (55,78)	93 (84,108)
tip prior	86 (71,104)	85 (73,101)	51 (32,78)	61 (44,88)	97 (82,117)

The tendency of the uncorrelated clock to lead to more uncertain estimates of divergence times compared with the autocorrelated clock can be understood in terms of the shrinkage power of the two alternative priors over rates. Uncorrelated models, because they assume that branch-specific rates are independent, can only detect the overall mean and variance of rates across branches, via their hyperparameters. If rate variation is substantial (as is the case here between small and large mammals), the prior expectation about the rate on any particular branch induced by the uncorrelated clock is then very diffuse. However, if there is long-range autocorrelation, then there is substantially more information to be gained about the joint distribution of branch-specific substitution rates. This information is entirely contained in the correlation between branches. Autocorrelated models are able to capture this correlation structure. As a result, they represent a more efficient shrinkage prior over branch-specific rates, compared with uncorrelated models, thus resulting in more informative posterior distributions over rates and, correlatively, over divergence times.

### (c) Mixed clock analyses using the node-dating approach

When applied to this placental dataset and in the node-dating settings, the mixed clock captures both an autocorrelated and uncorrelated component. Specifically, the Brownian component is inferred to contribute 61% of the total variance across branches, thus indicating that long-term autocorrelation accounts for a major part of rate variation. On the other hand, this still leaves a significant fraction (39%) of the total variation accounted for by the white noise process, suggesting that short-term fluctuations or, in any case, deviations from the Brownian assumption, are substantial. These inferred proportions are much larger than those obtained in the context of simulations under purely Brownian or purely white noise clocks, where at most 3% of the total rate variation is attributed the incorrect component (see above, section Simulations). This shows that the two pure clock regimes, either pure Brownian or pure white noise, are strongly rejected in the present case.

The divergence times inferred under the mixed clock (electronic supplementary material, figure S3) are more similar to those obtained under the white noise clock (mean Euclidean distance between median ages across all nodes of *d* = 2.3 Myr) than to those returned by the Brownian clock (*d* = 3.7 Myr). In particular, for those groups for which the estimates under the two pure clocks show the largest differences (Eulipotyphla, Euarchontoglires, Afrotheria and Placentalia, see above), the mixed clock essentially agrees with the white noise clock, within 2 Myr (tables 1 and 2). On the other hand, the average size of the posterior credible intervals is smaller under the mixed clock (12.1 Myr) than under the white noise clock (14.3 Myr), suggesting that, even if the mixed clock is not fundamentally different from the pure white noise clock in terms of point estimation, its autocorrelation component nevertheless allows for more information about divergence times to be collected from the global distribution of rates across branches.

Visual inspection of the inferred substitution rate history under the Brownian and the mixed clock gives interesting insight concerning how rates are partitioned by the mixed clock. The inferred history under the pure Brownian motion (electronic supplementary material, figure S4) is globally complex. It shows elevated rates in some rodents, insectivores, elephant shrews, the tenrec and hyrax as well as some bats, and globally low rates in elephants, sirenians, cetaceans and perissodactyls. Thus, the overall picture is congruent with the well-established negative correlation between substitution rate and body mass [12–15]. However, the patterns of rate variation are heterogeneous within each of these groups, in particular in Rodentia and in Chiroptera. In addition, isolated spikes of high rates of substitution can be observed at specific locations, in particular, at the base of Strepsirhini (Primates), Hystricomorpha (Rodentia), Yangochiroptera (Chiroptera) and Cetartiodactyla.

In contrast to this complex history of rate variation inferred under the pure Brownian clock, the Brownian component of the mixed clock (electronic supplementary material, figure S5) shows much simpler broad-scale patterns, essentially attributing high, medium or low rates across entire mammalian orders, in a way that seems to correlate with the typical body mass characterizing the members of these clades. For instance, according to this Brownian component, Rodentia or Eulipotyphla are globally fast-evolving, whereas Cetartiodactyla or Perissodactyla are globally slowly evolving, Carnivora or Primates showing an intermediate long-term substitution rate. The white noise component, on the other hand, reveals a few branches, at several key locations over the tree, with exceptionally high or low substitution rates (electronic supplementary material, figure S6). In particular, high substitution rates are inferred along the branches leading to Lorisidae in Strepsirhinii, to Hystricomorpha and to Yangochiroptera, as well as along several successive branches close to the base of Cetartiodactyla—thus in those regions of the tree where spikes of substitution rates are also detected by the pure Brownian clock (electronic supplementary material, figure S4). The white noise component detects higher rates in several other places, including on a couple of branches within Carnivora and Rodentia. Low rates, on the other hand, are inferred along the branches separating Chiroptera, Perissodactyla and Cetartiodactyla, as well as in several branches at the base of Rodentia. Altogether, the mixed clock is essentially capturing long-term patterns across placental mammals using a Brownian motion, while capturing the sharp and local deviations from this pattern using the white noise component.

### (d) Tip-dating analyses

The placental dataset was further analysed in a tip-dating context, using a modified version of the datasets of O'Leary *et al.* [35] and Meredith *et al.* [34]. Analyses were conducted without discrete morphological data, so as to analyse the patterns of molecular rate variation and to better characterize the behaviour of the mixed clock model in a simple case where only molecular sequences are considered.

One first major observation that can be made in this context is the strong sensitivity of divergence time estimation to the choice of the relaxed clock model (tables 1 and 2). On the one hand, the estimation under the white noise clock model (electronic supplementary material, figure S7) tends to give estimates that are globally younger than those obtained using the node-dating approach. The most important differences concern Perissodactyla, which is younger by 15 Myr, and Carnivora and Xenarthra, which are younger by approximately 10 Myr, compared to estimates obtained using the node-dating approach (tables 1 and 2).

By contrast, compared to the node-dating analysis, the log-Brownian clock (electronic supplementary material, figure S8) gives globally older ages, in particular concerning Primates, Rodentia, Eulipotyphla, as well as the four superorders and Placentalia (tables 1 and 2). The estimates under the Brownian clock are substantially older than those returned both by the white noise uncorrelated clock over the whole range of divergence times (*d* = 9.8 Myr between the two clocks). At the ordinal level, most orders are inferred by the Brownian clock to be older than under the white noise clock by at least 10 Myr, up to 20 Myr for Xenarthra, Afrotheria and Rodentia and 30 Myr for Primates (table 1).

These widely divergent estimates under the two relaxed clock models have very different implications in terms of the diversification scenario around the Cretaceous–Palaeogene (KPg) transition. Thus, the posterior mean number of lineages crossing the KPg boundary is 52 for the autocorrelated clock, versus 30 for the uncorrelated white noise clock. Except for the four orders belonging to Scrotifera (Carnivora, Cetartiodactyla, Perissodactyla and Chiroptera), which are inferred by the Brownian clock to be younger than or around the KPg boundary, the crown ages of the other placental orders shown in table 1 fall well within the Cretaceous (tables 1 and 2).

The white noise estimation would appear to be more congruent with current palaeontological knowledge, except perhaps for Perissodactyla, which would appear to be excessively young (between 37 and 52 Myr). The picture of early placental evolution suggested by the Brownian clock, in contrast, appears to be much less reasonable. This is particularly striking concerning the very old age inferred for Rodentia. Crown rodents first appear close to the Palaeocene/Eocene boundary (between 53 and 57 Ma; [36]), with only successively deeper stem members being found earlier in the Late Palaeocene and putatively in the Middle Palaeocene, which has a maximum age of 61.6 Myr. A far older crown rodent origin being missed by fossil sampling is even less likely in view of this stem to crown rodent transition in the fossil record being found in Asia, right where molecular biogeographic reconstructions place rodent origins [37].

A comparison between the prior and posterior credible intervals obtained under the two relaxed clocks is also instructive about how the priors on rates and on times interact in the present case. Here, a serial birth–death prior with diversified sampling is used as the prior on divergence times. This prior has a mechanistic interpretation in terms of species diversification and fossil sampling at constant rates through time and across lineages [31]. The prior credible intervals implied by this serial birth–death prior are relatively narrow (21.5 Myr on average across all nodes), thus indicating that this prior is quite informative.

**Figure 1.**
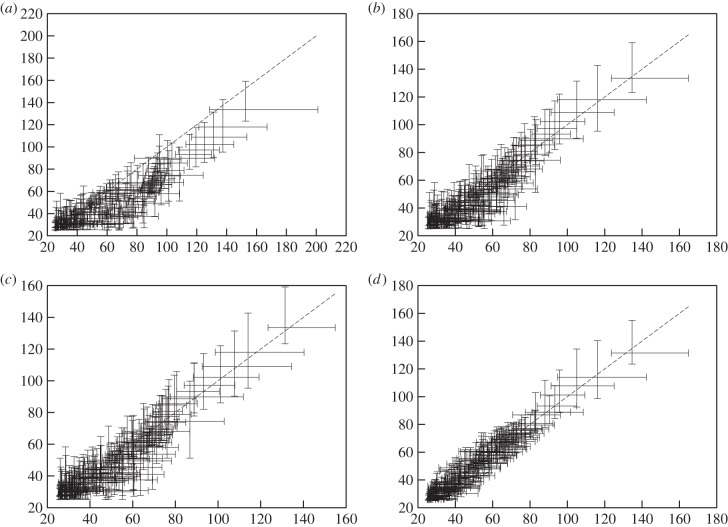
XY-plots comparing median divergence times and 95% credible intervals for the Brownian clock versus the serial birth–death prior (*a*), the white noise clock versus the prior (*b*), the mixed clock versus the prior (*c*), and the mixed clock versus the white noise (*d*).

Compared to this prior distribution, the posterior distribution under the Brownian clock is clearly shifted towards older ages (figure 1, mean quadratic distance of *d* = 13.1 Myr between prior and posterior median ages). By contrast, there is a closer match between the prior and posterior ages under the white noise clock (*d* = 6.4 Myr), in fact, closer than between the posterior median ages under the two alternative relaxed clock models (*d* = 9.8). At the level of orders and superorders, the largest deviations (greater than 10 Myr) between the prior and the posterior median ages under the white noise clock concern Primates, Rodentia and Eulipotyphla, which are younger, and Laurasiatheria older, under the posterior than under the prior distribution (table 1). The average size of the credible intervals is also slightly smaller under the Brownian clock (15.2 Myr) than under the white noise clock (16.7 Myr). These observations suggest that the regularization of rates across branches entailed by the Brownian clock is sufficiently strong to override the serial birth–death prior. By contrast, the white noise clock appears to be more labile. As a result, it can more easily yield to this informative prior on divergence times, by adapting its estimation of branch-specific rates accordingly.

Taken together, these observations show that, in the context of the present tip-dating analysis, the estimation of divergence times is strongly sensitive to the specific relaxed clock model used for conducting the inference. They also show that, in the absolute, the Brownian relaxed clock is less flexible and thus more informative than the white noise clock. However, the globally unreasonable ages obtained under the Brownian clock suggest that this rate prior may in fact be too rigid, thus leading to biased estimation of divergence times.

### (e) Mixed clock analyses using the tip-dating approach

If the mixed clock is used in the context of the tip-dating analysis, the white noise component accounts for 46% of the variation in the substitution rate across branches. This estimate is congruent with the results obtained in the context of the node-dating analysis (40%), which confirms that rate variation across placental mammals, in spite of having a strong autocorrelated component, nevertheless displays strong deviations from the pure Brownian assumption.

**Figure 2.**
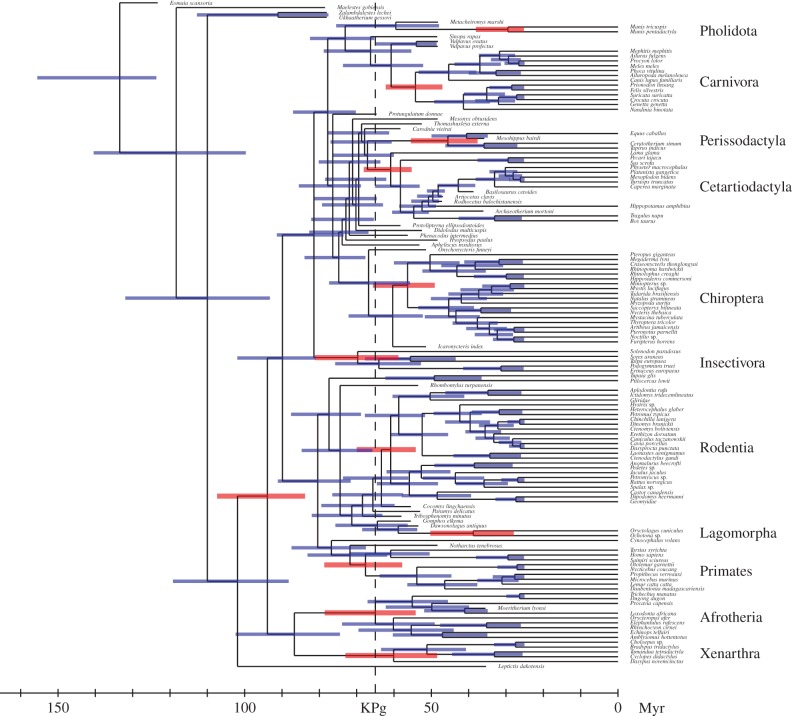
Inferred divergence times under the mixed clock, using the tip-dating approach (posterior median, coloured bars: 95% credible intervals; red bars: Placentalia, as well as orders and super-orders indicated to the right).

**Figure 3.**
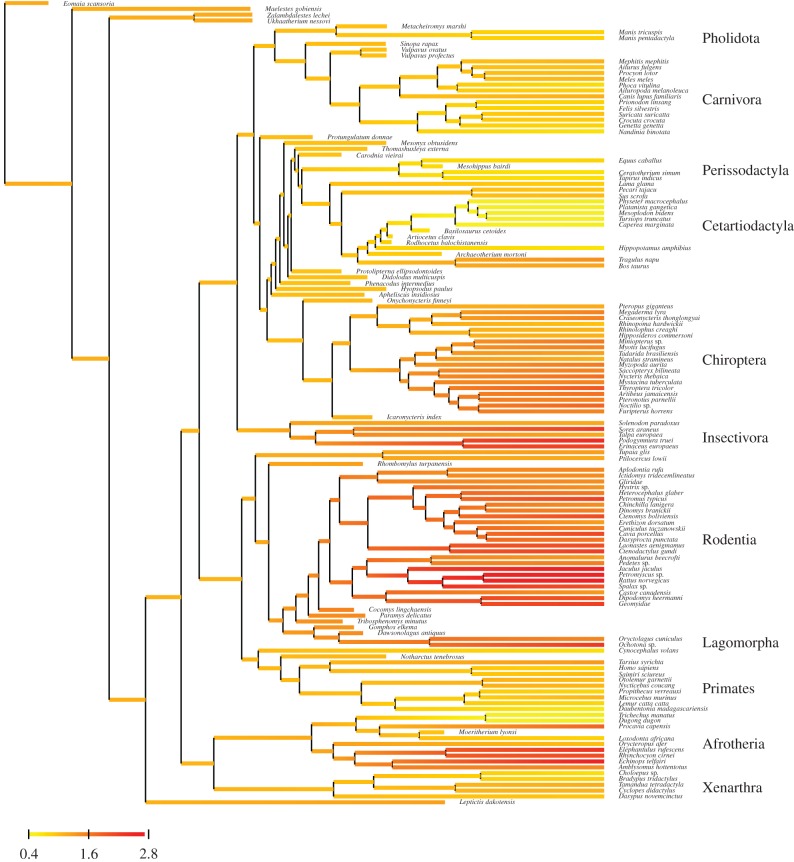
Inferred history of long-term rate variation (Brownian component of the mixed clock) using the tip-dating approach.

Strikingly, however, although half of the total rate variation inferred over the tree is attributed to the autocorrelated component, divergence times inferred under the mixed clock (figure 2) are much more similar to those estimated under a pure uncorrelated clock (*d* = 3.3, electronic supplementary material, figure S7) than to those inferred under a pure Brownian clock (*d* = 9.2, electronic supplementary material, figure S8). In addition, as in the case of the pure white noise clock, the posterior median ages under the mixed clock are similar to the prior median ages (*d* = 6.1 between prior and posterior median ages, versus *d* = 6.4 under the white noise clock and *d* = 13.1 under the Brownian clock; figure 3). The size of the credible intervals under the mixed clock (15.6) is intermediate between those obtained under the Brownian clock (15.2) and those returned by the white noise clock (16.7).

A possible interpretation of these observations would be as follows. Like the pure Brownian clock, the mixed clock is able to detect the long-range autocorrelation signal. However, unlike the Brownian clock, it does not strongly rely on those long-range effects to inform divergence time estimation. Instead, the mixed clock essentially opts out whenever the underlying informative prior on divergence times contradicts its long-range tendencies, in which case its white noise component acts as a buffer. As a result, and like what happens under the pure white noise clock, the estimation of divergence times is more strongly driven by the serial birth–death prior.

Interestingly, at the ordinal level, the main differences between the pure white noise and the mixed clock seem to correlate with body size. Under the mixed clock, the crown age of orders of large and long-lived mammals (Primates, Carnivora, Perissodactyla and Cetartiodactyla) tends to be older, whereas the crown age of orders of small mammals (Rodentia, Eulipotyphla, Chiroptera) tends to be younger, than under the white noise clock (table 1). Thus, the autocorrelated component of the mixed clock does appear to have some influence on the dating analysis. This influence is most pronounced in the case of Rodentia, whose crown age is inferred by the mixed clock to be well within the Palaeogene, at 60 Myr (54,70), thus in good agreement with current palaeontological evidence (see above). For the other placental orders, the influence of the autocorrelated component appears to be rather marginal.

**Figure 4.**
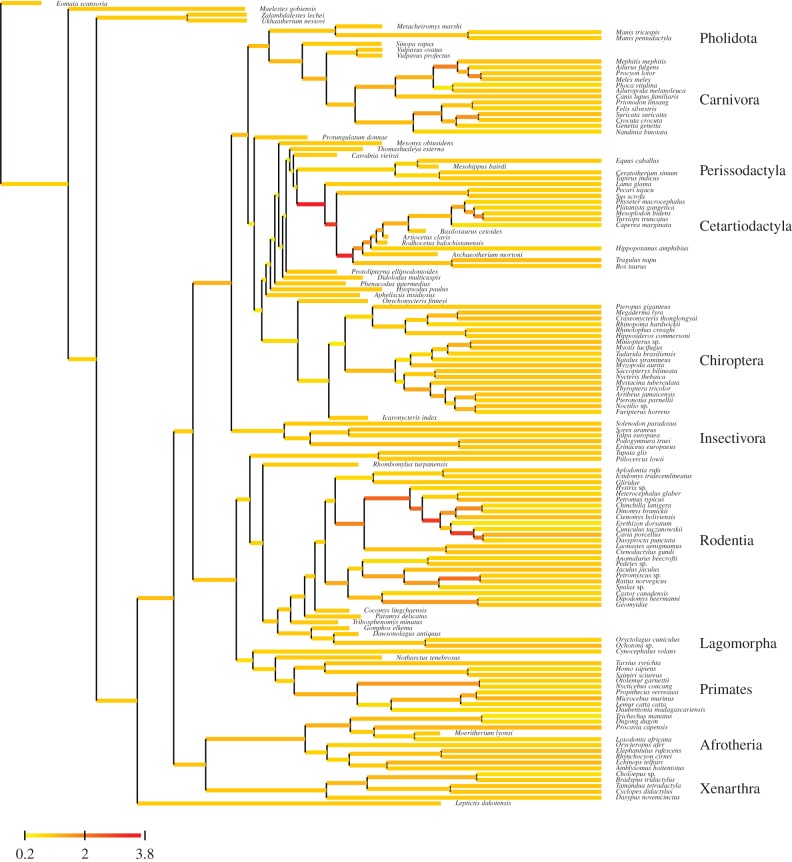
Inferred history of short-term rate variation (white noise component of the mixed clock) using the tip-dating approach.

Visual inspection of the estimated substitution rate history along the tree under the two relaxed clock models, either pure Brownian (electronic supplementary material, figure S9) or mixed (figures 3 and 4), confirm previous observations gathered in the context of the node-dating analysis (electronic supplementary material, figures S4–S6). The Brownian component of the mixed clock returns a globally smooth history of rate variation over the tree, capturing broad patterns that would seem to correlate well with large-scale trends in body-size evolution across placental mammals, whereas the white noise component detects a few strong but isolated departures of substitution rate. The sets of branches showing strong deviations from the pure Brownian assumption are overlapping between the tip-dating and the node-dating analyses, especially the high rates inferred in the early region of crown Cetartiodactyla and in Hystricomorpha.

Altogether, and as was observed in the node-dating analysis, the use of a mixed clock in the context of the tip-dating analysis reconciles the most important features classically obtained under each of the pure clocks. By absorbing short-term effects and/or sudden deviations through its white noise component, the mixed clock reveals more clear-cut long-term trends which appear to correlate with global patterns of life-history evolution. Simultaneously, it leads to more recent and more reasonable estimates for the divergence times of the ancestors of placental orders and superorders, similar to those obtained under the uncorrelated clock, although with some marginal input contributed by the autocorrelated component.

## 3. Discussion

### (a) Mixing autocorrelated and uncorrelated clocks

In this work, a mixed relaxed clock model was introduced, which can be mechanistically interpreted as a rate variation process undergoing short-term fluctuations on the top of Brownian long-term trends. Statistically, this mixed clock represents an alternative solution to the problem of choosing between autocorrelated and uncorrelated relaxed clocks, by proposing instead to combine their respective merits, thus simultaneously capturing the long-term autocorrelation signal while accommodating the local deviations. This mixed relaxed clock was applied to a dataset of 105 extant placental mammals, using two alternative dating paradigms: node-dating, with a rich set of fossil constraints and tip dating.

In the case of the node-dating analysis, divergence times are relatively well constrained by fossil calibrations. These settings therefore provide an experimental set-up in which patterns of rate variation across the tree can be assessed relatively independently of how rates in turn could influence the global distribution of divergence times. In this context, substantial deviations of the molecular rate variation over the tree from a pure Brownian model are detected.

These deviations from the pure Brownian autocorrelated model are also found in the context of a tip-dating analysis. In this context, the choice of the relaxed molecular clock model appears to have a strong influence on the estimation of divergence times. Comparing the two analyses, node- and tip-dating, with each other and with palaeontological data globally suggests that the classical Brownian (lognormal) autocorrelated model is globally inadequate, overly rigid, and as a result, leads to biased estimation of divergence times. By contrast, the use of a mixed clock model appears to lead to globally more reasonable date estimates.

### (b) Towards more elaborate models of rate evolution

The fundamental causes behind the deviations detected here by the mixed relaxed molecular clock are not totally clear. Several possible explanations could be considered, which are not mutually exclusive.

First, some molecular evolutionary phenomena are known to induce transient fluctuations in the substitution rate. In particular, recombination-induced biased-gene conversion has been suggested as one particularly important source of local and transient bursts of substitutions along the genome [24]. Such transient phenomena should be relatively well captured by a white noise process, even if they should ideally be modelled as gene-specific effects [25]. If dating analyses are conducted separately on each gene of the concatenation (not shown), some gene-specific differences in the patterns of rate deviation are indeed detected by the white noise component of the mixed clock. However, the branches showing the most extreme deviations (in particular, in the early part of Cetartiodacyla and at the base of Lorisiforma and of Hystricomorpha) are recovered uniformly across all genes. Given current views about molecular evolution in mammals, genome-wide transient deviations are perhaps more difficult to invoke in the present case than locus-specific deviations.

In some cases, the white noise process may be absorbing rate deviations induced by incomplete lineage sorting (ILS, [38,39]). The discrepancies among gene trees are not properly accounted for by the supermatrix approach used here, and it is possible that gene-species tree discrepancies result in a systematic and artefactual displacement of ancestral substitutions towards the more recent branches of the ILS-affected region. In particular, exceptionally low rates are inferred along the branches separating Chiroptera, Perissodactyla and Cetartiodactyla, as well as in the branches separating Hystricomorpha, Sciuromorpha and Castoriomorpha. In these two cases, ILS has been suspected [40]. However, ILS at best accounts for only a fraction of the deviations detected here, most of which are not located in regions particularly suspected to be affected by strong ILS. Mixed clock approaches could be used in the context of explicit gene-species reconciliation models [41–43].

In principle, some of the deviations observed here could be artefacts caused by incorrect fossil calibrations. The fact that the same branches tend to be affected in the two contexts, node- and tip-dating, suggests that most of the deviations detected in the present analysis are not the result of such mis-specifications of fossil constraints. Still, some of the deviations observed here are clearly dependent on specific calibrations. In particular, a very high rate is detected under the node-dating approach along the branch leading to Lorisiformes (*Otolemur* and *Nycticebus*; electronic supplementary material, figure S6). Lorisiformes are constrained by a lower calibration at 37 Myr (based on the *Saharagalago* fossil), and the crown age of this group is inferred to be very close to this lower bound (between 37 and 42 Myr). In the tip-dating analysis, the age of Lorisiformes is estimated to be much younger (between 26 and 32 Myr), and correlatively, the rate deviation detected along the stem branch leading to Lorisiformes is much milder than in node-dating context. There are two possible interpretations of this particular observation. On the one hand, the fossil calibration could be correct, and the rate shift inferred on that branch could be real. The fact that Lorisiformes are among the smallest- and shortest-lived primates suggests at first sight that this particular rate deviation could correspond to a case where a rapid shift has indeed occurred in the life-history strategy, leading to a shorter generation time. However, in that case, the higher substitution rate should have been maintained over the subsequent branches, within Lorisiformes. Instead, what is observed here is a high rate only along the basal branch of the group (electronic supplementary material, figure S4). Thus, alternatively, the rate shift could be an artefact owing to incorrect calibration. In favour of this interpretation, the placement of the *Saharagalago* reference fossil as a crown Lorisiformes [44], which defines the lower bound at 37 Myr, was not recovered in another study, Marivaux *et al.* [45], where *Saharagalago* was instead inferred to be a stem Lorisiformes.

Regardless of the validity of the fossil calibration, the case of Lorisiformes illustrates the need to make a distinction between sudden and transient jumps in the rate of substitution. Transient rate jumps, by definition, are expected to rapidly revert to the substitution rate prevailing before the jump. Local and transient episodes of biased-gene conversion, caused by short-lived recombination hot-spots [24,25], represent good examples of such transient phenomena. Sudden jumps in contrast, can result in changes in the rate of substitution that can be maintained for an arbitrarily long time after the jump. These jumps could be caused by rapid shifts in ecological or life-history strategies, which would result in a correlative change in the generation time, or by the loss of specific genes involved in DNA repair. More complex patterns of sudden rate variation could be imagined. For instance, in Cetartiodactyla, a rapid and parallel decrease in substitution rate across the group, from a small and fast-evolving ancestor, has been suggested [22], which appears to result in violations of the pure Brownian assumption [14].

In theory, the mixed clock introduced here is meant to detect only transient jumps. However, the white noise component of the mixed clock might also be able to buffer against the impact of sudden rate shifts, by locally absorbing the deviation. This could explain the pattern observed here in Cetartiodactyla, where the white noise component infers high rates of evolution along several successive branches in the early part of the phylogeny of the order (figure 4; electronic supplementary material, figure S6). These high rates estimated by the white noise component would compensate for the fact that, because of its strong inertia, the Brownian component tends to over-extrapolate the low-rate signal contributed by extant Cetartiodactyla too far up in the tree. In other words, in the presence of sudden rate shifts, the mixed clock would bring some warranted flexibility, compared to the pure Brownian clock—although in a way that does not really correspond to its intended mechanistic meaning.

Ultimately, the mixed clock considered here may thus not represent a correct formalization of the macro-evolutionary processes underlying rate variation. Alternative models should probably be considered, such as those already used in the context of the comparative method for describing the evolution of quantitative traits. In this direction, models of burst and stasis [46], possibly associated with adaptive radiations, or stochastic processes allowing for large deviations [47,48] represent promising directions for further methodological developments. Of note, such models allowing for rapid shifts in the rate of substitution could also be mixed with an additional white noise layer (possibly on a gene-specific basis), accounting for the residual transient effects which are known to occur.

In any case, even if it does not yet represent a convincing model of rate evolution, the mixed clock introduced here already allows for a simple quantitative test of how much of the total rate variation over the tree is not correctly captured by the standard autocorrelated lognormal clock. Simultaneously, it offers a simple visualization of the hot-spots of non-Brownian rate variation over the tree. From there, further elaboration of the relaxed clock model, in a mechanistic and process-oriented perspective, can then be considered.

### (c) The rate-versus-time dilemma

From a statistical perspective, Bayesian molecular dating can be seen as a trade-off between two regularization priors, over times and over rates. The tip-dating analysis conducted here gives interesting insights about how these two priors interact in practice. In the end, however, the observations gathered in this analysis lead to the following dilemma. On the one hand, the pure Brownian clock provides an inadequate description of rate variation through time and, in particular, tends to over-regularize the distribution of rates across branches. This over-smoothing tendency appears to lead to strong biases in the estimated dates. On the other hand, if short-term variation in the substitution rate is accommodated by introducing an uncorrelated component, the relaxed clock loses much of its regularizing power. As a result, the molecular dating analysis becomes more strongly determined by the prior on divergence times. If this prior happens to be inadequate, in particular if it underestimates the true extent of the variation in diversification rates over time and among groups, then it will over-regularize the distribution of times across nodes—thus also resulting in biases in the estimated dates.

In the present case, there are good reasons to believe that the homogeneous serial birth–death prior is indeed over-regularizing the distribution of divergence times. This prior assumes uniform speciation, extinction and fossil sampling rates over time and across the whole tree. Yet, real diversification processes in eutherians are certainly much more varied, being characterized by a major ecological release right after the KPg mass-extinction [49], as well as a complex biogeographic history [37]. Furthermore, diversification and fossilization rates are probably very different across ecologically disparate mammalian orders. This complex diversification history may have resulted in large-scale patterns, which have probably been smoothed out by the use of a homogeneous prior. Thus, it is not clear how much confidence should be put in the overall picture of placental evolution obtained here, even under the mixed relaxed clock.

In the face of this dilemma, we are left with two possible choices. In one direction, further elaboration on the side of the relaxed clock models could and should probably be considered. Possibly, more refined models, that would correctly account for departures from the Brownian assumption, although without being as flexible as the mixed clock introduced here, could result in relaxed clock models that would still have a sufficient regularization power to lead to informative and robust estimation of divergence times, thus making the choice of the prior on divergence times less critical. However, if true patterns of rate variation are sufficiently extreme, then it is possible that even the empirically most accurate relaxed clock model will turn out to be inherently weak in its regularization ability.

Adding more loci to the analysis could in some cases increase the regularizing power of the molecular clock. However, this will be the case only if transient jumps and other short-term effects are locus-specific and not strongly correlated among loci. In this situation, the variance contributed by the white noise component of the mixed clock is expected to vanish as more and more loci are considered. On the other hand, adding more loci will not really help if both the short- and long-term variations in the substitution rate are global, genome-wide effects.

Using morphological data to calibrate the morphological clock, in a total-evidence framework, could bring additional information further constraining the estimation of divergence times [29,30,50–52]. However, morphological evolution may be even more erratic than molecular evolution. In particular, morphological evolution is directly influenced by strong selective forces induced by the ecological environment or by sexual selection, all of which are expected to undergo substantial fluctuations over short evolutionary timespans. As a consequence, the ability of morphological characters to directly inform divergence times through the morphological clock might be limited in practice. On the other hand, morphological characters are likely to be a very useful source of information for placing fossils in the tree topology in a total-evidence framework [29,30].

Alternatively, if it turns out that both the molecular clock and its morphological counterpart are intrinsically affected by short-term effects, such that their regularization power is inherently limited, then the inference will be fundamentally determined by the prior on divergence times. However, this means that more effort should be devoted to the design of species diversification models that would be empirically more adequate than the models currently used. Such models could possibly be informed by the palaeontological record, which, beyond the local calibrations induced by specific fossils, also provides much information about the evolution of species richness through time and about the biogeography of the clade under study.

In any case, on a large evolutionary scale such as that represented by placental mammals, characterized by extensive rate variation, estimation of divergence times should perhaps not just be seen as classical molecular dating problem, in which the molecular clock is supposed to play the central role. Instead, it should more fundamentally be seen as an integrative dating problem, in which patterns of speciation and extinction are also a major component of the analysis.

## 4. Methods

### (a) Data, taxon sampling, phylogeny and fossil calibrations

The nucleotide dataset of Meredith *et al.* [34] was first restricted to placental mammals and then reduced to 105 taxa in a way that was intended to better correspond to the diversified sampling assumption. Specifically, a cut-off time of 25 Myr was chosen. Then, based on the time-calibrated phylogeny published by Meredith *et al.* [34], only one terminal taxon was chosen for each lineage crossing the cut-off time. For the tip-dating analyses, the 105 taxon dataset was combined with the set of eutherian fossils of the analysis of O'Leary *et al.* [35], excluding *Hapalops*, which is younger than 25 Myr. This resulted in a total of 138 eutherian species. Altogether, this taxon sampling can be seen as approximating a uniform fossil sampling rate between the origin of Eutheria and 25 Myr, followed by a fossil sampling rate of 0 between 25 Myr and the present.

For both node- and tip-dating analyses, the phylogeny was constrained *a priori*. For the node-dating analyses, the phylogeny of extant placental mammals was constrained based on the nucleotide-based phylogeny of Meredith *et al.* [34]. For the tip-dating analyses, the phylogeny was obtained as follows. First, the phylogeny of Meredith *et al.* [34] was used as the backbone for the phylogeny spanning extant taxa. Then, restrictions about possible fossil placements were defined based on expert knowledge, in the form of a series of clade constraints (electronic supplementary material, table S5). Finally, a Bayesian total-evidence analysis [30] was conducted under the white noise clock, under all of the constraints defined above and using the morphological character matrix of O'Leary *et al.* [35]. All this was done using the RevBayes programming environment [53]. The posterior consensus tree of two independent analyses was calculated, which was then used as the fixed tree topology for all remaining analyses presented in this article.

To conduct the constrained total-evidence analysis, the matrix of morphological characters of O'Leary *et al.* [35] was extended with missing entries so as to encompass the 138 extant taxa considered here. The resulting character matrix was partitioned into several components, based on the number of distinct states represented in each column of the data matrix. Only the components corresponding to 2, 3 and 4 distinct states were used. Together, they account for 3212 out of the 4541 characters of the original data matrix. For each component, a Jukes–Cantor model was used, with the adequate number of states. Note that the likelihood was not corrected for unobserved site-patterns [54], as this option was not available in RevBayes at the time of the analysis. The prior constraints on fossil placement were used for two reasons: first, to restrict the search space and, second, to override some of the problematic fossil placements observed in the unconstrained analyses using the morphological character matrix of O'Leary *et al.* [35].

For the node-dating analyses, all of the fossil calibrations used by Meredith *et al.* [34], which are still valid under the present taxon subsampling, were used. They are reported in the electronic supplementary material, table S6. They were implemented as hard bounds. For the tip-dating analysis, uncertainty about fossil ages was accounted for, thus addressing the potentially important problem raised by O'Reilly *et al.* [52]. Allowing for uncertainty about fossil ages was implemented by allowing the tips corresponding to fossils to move during the Markov chain Monte Carlo (MCMC), within the intervals defined in the electronic supplementary material, table S7. As a result, the likelihood is summed over all possible serially sampled time-calibrated phylogenies that are compatible with these interval constraints.

### (b) Serial birth–death with diversified sampling

For the tip-dating analyses, a birth–death process with uniform serial sampling [31] and diversified sampling of extant taxa was used as the prior on divergence times. This birth–death process assumes constant rates of speciation *λ*, extinction *μ* and fossil sampling *ψ*, between the time of origin of the process 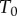 and a cut-off time 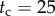 Myr. Neither cladogenesis nor any fossil sampling is allowed between *t*_c_ and the present. The ancestral lineages (i.e. surviving up to the present time) arriving at the cut-off time of 25 Myr are assumed to give rise to the total diversity of extant placental mammals, which was here considered to be equal to 5000 species. The probability density of a phylogeny under this process has been derived previously [55], except for the fact that, here, fossil sampling is always coupled with extinction (as in [31]), and thus, all sampled fossils are tips of the phylogeny. By contrast, the fossilized birth–death process [32], combined with diversified sampling [55], allows for some of the sampled fossils to be ancestral to other fossils or to extant taxa. However, in the present case, fossil sampling is very sparse compared with the total diversity of the clade, and as a result, the probability of sampling a fossil that will be ancestral to other represented lineages in the tree is very low (of the order of 0.001). In this regime, the tip-only approximation used here is expected to be accurate.

### (c) Mixed clock models

Consider a phylogenetic tree *Ψ*, with *P* tips. The tree is time-calibrated but not necessarily ultrametric (in the tip-dating case, tips are serially sampled). In the following, a forward-in-time parametrization is used. The branches of the tree are indexed by 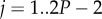, with branch *j* beginning at time 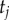 and ending at time 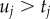.

The rate of nucleotide substitution is theoretically assumed to be a continuous-time process, generically denoted as *r*(*t*). The logarithm of the rate is noted 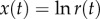. Because the rate is defined along a tree, thus having different values at the same time on different branches, a more specific notation will be used below, with 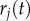 denoting the instantaneous rate on branch *j* at time *t*.

Of interest in the context of models of sequence evolution is the average rate over each branch of the phylogenetic tree. For branch *j*, this averaged rate is defined as

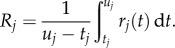



Multiplying the average rate by the duration of the branch gives the effective branch length 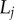, measuring the expected number of substitutions per site along that branch:

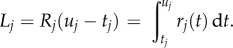



The Brownian and white noise clock models have been introduced elsewhere [2,5]. Briefly, the white noise model assumes that the instantaneous rate of substitution follows a white noise process, whose stationary distribution is a gamma distribution of mean 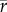 and variance denoted here as 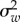. Under the white noise model, the average rate over branch *j* is gamma distributed, of mean 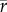 and variance inversely proportional to the duration of the branch [2]:

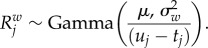



Under the Brownian model, the logarithm of the instantaneous rate, *x*(*t*), follows a Brownian process of variance per unit of time 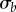. Given the value of *x* at time 0, *x*(0), the value *x*(*T*) at time *T* is normally distributed of mean *x*(0) and variance 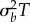. The Brownian process is Markovian, and therefore, the probability density of a configuration of *x* at all nodes of the phylogenetic tree can be obtained as the product of independent normal distributions. Conditional on the values of the rate at both ends of branch *j*, 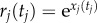 and 
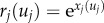
, the mean rate induced by the Brownian clock over the branch is not analytically available. As often done [5], it is here approximated by simply taking the arithmetic average of the rates at both ends:

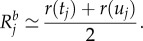



The mixed clock is a simple multiplicative combination of the white noise and the Brownian clock introduced above. Theoretically, the mixed clock can be seen as an additive combination of the instant values of logarithm of the rates under the white noise and the Brownian clocks:
4.1
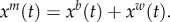



Computationally, the mixed clock was simply implemented by multiplying, for branch *j*, the rates given by the white noise and the Brownian clock:
4.2
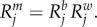



Multiplying this rate with the duration of the branch then gives the effective substitutional length of the branch under the mixed clock:

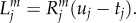



Note that equation (4.2) represents a rather crude approximation of the continuous-time process defined by equation (4.1). This approximation amounts to assuming that the Brownian clock has constant rate 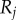 along branch *j*. Ultimately, more accurate implementations of the mixed clock, as well as of the Brownian clock, could be developed, using fine-grained discretization schemes such as developed by Horvilleur & Lartillot [56]. However, in practice, the Brownian component of the mixed clock is slowly varying, and therefore, this approximation should give reasonable results in the present context.

### (d) Substitution model

The nucleotide sequences were assumed to evolve according to a general time-reversible process, using the standard parametrization in terms of relative exchange rates and equilibrium frequencies. Neither variation across sites nor across partitions was accommodated. Preliminary runs, using the node-dating formalism already implemented in PhyloBayes [57], with and without rate variation among sites, resulted in very similar divergence times estimation (mean euclidean distance of 0.8 Myr between the vectors of posterior mean divergence times between the two analyses, maximum deviation of 4 Myr across all nodes), suggesting that accounting for varying rates across sites is not critical in the present context.

### (e) Priors

The following priors were used. For the node-dating analyses, a birth–death prior with species sampling was used. This prior has three parameters: the birth rate (*λ*), the death rate (*μ*) and the sampling fraction *ρ*. Only two of these three parameters are identifiable, which we define as 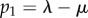 and 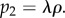 An exponential prior of mean 1 was used for 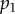 and 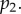 An exponential of mean 100 was used for the age of the root of the tree. For the tip-dating analyses, we used exponential priors of mean 1 for the diversification rate 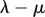, and the fossil sampling rate *ψ*, of mean 10 for the extinction rate *μ*, and of mean 100 for the age of the root. For the clock models, we used exponential priors of mean 1 for the mean and the variance of the white noise process (in the case of the mixed clock, the white noise process is constrained to be of mean 1). For the Brownian clock, we used a truncated Jeffreys prior, between 10^−3^ and 10^3^ for *σ^b^* and a truncated uniform prior between −100 and 100 for *x*(0), the logarithm of the substitution rate at the root of the tree. For the substitution process, the two vectors of relative exchange rates and equilibrium frequencies were both endowed with uniform Dirichlet distributions.

### (f) Implementation

The models described above were implemented using the previously developed Coevol program [14,20,25]. This programming environment uses an explicit graphical model representation [53], which makes the development of new models, such as the mixed clock introduced here, relatively straightforward. The detailed MCMC algorithms used in this program are described by Lartillot & Poujol [20]. Briefly, an MCMC cycle starts by sampling a detailed substitution history for all sites and over the whole tree from the conditional posterior distribution defined by the current parameter configuration. This stochastic mapping of substitution events [58] is technically a data augmentation, for which compact sufficient statistics (total number of substitution events between each possible pair of nucleotide states, total time spent in each state) can be calculated separately for each branch and summed over all sites. Conditional on these sufficient statistics, the parameters of the model (rates, times, substitution model parameters and hyperparameters), are then updated in succession, using standard Metropolis–Hastings updates. A large number of such updates (typically several hundreds for each parameter) are conducted during each cycle. This alternation between Gibbs resampling of the substitution histories and Metropolis updates of the parameters conditional on the substitution histories represents a computationally efficient approach for sampling from the posterior distribution over the model parameters [59].

In the context of this implementation, the main additional algorithmic developments for the present needs were the re-implementation of the serial birth–death prior with diversified sampling, as well as one additional MCMC move specifically for the mixed clock model. This move is a compensatory move that simply draws a uniform random variate *m* centred on 0, adds *m* to all instant log-rate values across nodes defined by the Brownian component and divides all rates under the white noise component by 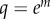. This move leaves the sequence likelihood unchanged (rates under the Brownian motion being increased by a factor *q*, exactly compensating the decrease by a factor *q* incurred by the rates under the white noise component). The Hastings ratio of this move is equal to 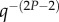 (where 2*P*−2 is the number of degrees of freedom of the white noise component).

The MCMC was run for a series of approximately 21 000 cycles, removing the first 1000 cycles and using the last 20 000 cycles to approximate a sample from the posterior distribution. The tracecomp program, from the PhyloBayes package, was used to assess effective sample size (which was greater than 100 in most cases) and the relative discrepancy between posterior mean estimates (smaller than 0.1 in most cases) for key summary statistics (logarithm of the prior density and the likelihood, total length of the timetree, hyperparameters of the prior on rates and times).

The implementation is available on www.phylobayes.org (in the Coevol software package).

### (g) Simulations

In the case of node-dating, simulations were conducted using the posterior predictive formalism. Specifically, the MCMC sampler was run on empirical data under each of the three models (pure Brownian, pure white noise or mixed clock). In a second step, for each run, 10 regularly spaced points from the MCMC (burn-in excluded) were used as parameter configurations for simulating 10 replicates of the original dataset. This resulted in a total of 30 simulation replicates (10 under each relaxed clock model). These simulated data were then re-analysed under the mixed clock model. For each replicate, the posterior mean percentage of variance contributed by the white noise component was estimated and compared to the true value. The true value is trivially 0% for the pure Brownian model and 100% for the pure white noise model. In the case of the mixed model, the true value depends on the parameter configuration that was used to obtain the posterior predictive simulation draw. It was calculated directly from this parameter configuration, before simulating the data (reported in the electronic supplementary material, tables S1–S4).

In the case of tip-dating, three series of simulations were conducted. A first series used parameter configurations drawn from the posterior distribution for all parameters, except for divergence times which were drawn from the prior distribution (serial birth–death model). Draws from the serial birth–death prior were obtained by running a separate MCMC under the prior, and then taking the configuration of divergence times at 10 regularly spaced points from the resulting chain. These configurations were recombined with the 10 parameter configurations from the posterior distribution. A second series of simulations were conducted, which was identical to the first series, except that the extinction rate was constrained to be equal to 0 and the diversified sampling option was deactivated during the run under the prior, thus emulating a Yule prior without diversified sampling. A final series of simulations was conducted using the posterior predictive formalism, as in the case of the node-dating analysis. All these 90 replicates were re-analysed under the mixed clock model, using the serial birth-death prior. Thus, the first series of simulations is under matching prior over divergence times, the second under mis-specified prior over divergence times and the third is most faithful to the empirical data.

To reduce the computational burden, the whole simulation experiment was conducted based on a reduced version of the original dataset, using only the BRCA2 partition (5046 aligned nucleotide positions) for the complete set of 105 taxa.

## Supplementary Material

Data

## Supplementary Material

Supplementary material

## Supplementary Material

Source code
