# Supplementary material for: A mixed relaxed clock model
Source: Philos Trans R Soc Lond B Biol Sci. 2016 Jul 19;371(1699):20150132. doi: 10.1098/rstb.2015.0132 (PMC4920333; doi:10.1098/rstb.2015.0132)
Supplement: Supplementary material [file rstb20150132supp2.pdf]

February 18, 2016

A mixed relaxed clock model. Supplementary material

Nicolas Lartillot[1], Matthew J Phillips[2], Fredrik Ronquist[3].

[1] *Laboratoire de Biométrie et Biologie Evolutive, Lyon, France.*

nicolas.lartillot@univ-lyon1.fr

[2] *School of Earth, Environmental and Biological Sciences, Queensland University of Technology, Brisbane, Australia.*

m9.phillips@qut.edu.au

[3] *Department of Biodiversity Informatics, Swedish Museum of Natural History, Box 50007, SE-104 05 Stockholm, Sweden.*

fredrik.ronquist@nrm.se

Running head: Mixed clock

Keywords: Molecular dating, Relaxed molecular clock, Bayesian inference, Monte Carlo.

Supplementary Tables

Table S1: Simulation results under the node-dating settings: true versus inferred proportion of variance contributed by the whitenoise component. Each pair of entries corresponds to a replicate simulated with parameter configurations drawn from the posterior distribution under the model indicated on the top line.

Brownian		whitenoise		mixed	
true	inferred	true	inferred	true	inferred
0%	3%	100%	97%	31%	26%
0%	4%	100%	99%	19%	28%
0%	2%	100%	98%	31%	27%
0%	3%	100%	96%	27%	24%
0%	2%	100%	97%	35%	37%
0%	2%	100%	99%	30%	38%
0%	2%	100%	99%	34%	16%
0%	2%	100%	99%	31%	53%
0%	2%	100%	99%	41%	30%
0%	2%	100%	99%	24%	15%

Table S2: Simulation results under the tip-dating settings: true versus inferred proportion of variance contributed by the whitenoise component. Each pair of entries corresponds to a replicate simulated with parameter configurations drawn from the posterior distribution under the model indicated on the top line, except for divergence times, which were drawn from the serial birth-death prior with diversified sampling.

Brownian		whitenoise		mixed	
true	inferred	true	inferred	true	inferred
0%	3%	100%	99%	47%	40%
0%	3%	100%	99%	40%	31%
0%	4%	100%	99%	38%	71%
0%	2%	100%	99%	42%	64%
0%	3%	100%	98%	37%	60%
0%	3%	100%	99%	50%	60%
0%	5%	100%	99%	45%	55%
0%	5%	100%	98%	47%	38%
0%	12%	100%	99%	36%	43%
0%	8%	100%	99%	35%	52%

Table S3: Simulation results under the tip-dating settings: true versus inferred proportion of variance contributed by the whitenoise component. Each pair of entries corresponds to a replicate simulated with parameter configurations drawn from the posterior distribution under the model indicated on the top line, except for divergence times, which were drawn from a serial Yule prior without diversified sampling.

Brownian		whitenoise		mixed	
true	inferred	true	inferred	true	inferred
0%	13%	100%	99%	47%	85%
0%	13%	100%	99%	40%	98%
0%	58%	100%	99%	38%	87%
0%	27%	100%	99%	42%	71%
0%	20%	100%	99%	37%	90%
0%	13%	100%	99%	50%	68%
0%	41%	100%	99%	45%	78%
0%	17%	100%	99%	47%	38%
0%	14%	100%	99%	36%	65%
0%	20%	100%	98%	35%	81%

Table S4: Simulation results under the tip-dating settings: true versus inferred proportion of variance contributed by the whitenoise component. Each pair of entries corresponds to a replicate simulated with parameter configurations drawn from the posterior distribution under the model indicated on the top line.

Brownian		whitenoise		mixed	
true	inferred	true	inferred	true	inferred
0%	11%	100%	97%	47%	19%
0%	45%	100%	99%	40%	60%
0%	14%	100%	92%	38%	28%
0%	9%	100%	99%	42%	49%
0%	18%	100%	99%	37%	48%
0%	18%	100%	99%	50%	45%
0%	16%	100%	95%	45%	65%
0%	25%	100%	99%	47%	44%
0%	14%	100%	99%	36%	26%
0%	28%	100%	98%	35%	34%

Table S5: Clade constraints used for estimating the tree topology for the tip-dating analysis.

Metacheiromys marshi	stem Pangolin
Moeritherium lyonsi	stem Elephant
Notharctus tenebrosus	Primates (stem or crown)
Dawsonolagus antiquus	stem Lagomorpha (1)
Gomphos elkema	stem Lagomorpha (2, i.e. older than Dawsonolagus)
Paramys delicatus	stem Rodentia
Cocomys lingchaensis	stem Rodentia
Tribosphenomys minutus	stem Rodentia
Rhombomylus turpanensis	stem Glires or Lagomorpha or Rodentia
Didolodus multicuspis	stem Cetartiodactyla + Perissodactyla
Thomashuxleya externa	stem Cetartiodactyla + Perissodactyla
Protolipterna ellipsodontoides	stem Cetartiodactyla + Perissodactyla
Carodnia vieirai	stem Cetartiodactyla + Perissodactyla

Table S5 (continued): Clade constraints used for estimating the tree topology for the tip-dating analysis.

Vulpavus ovatus	stem Carnivora
Vulpavus profectus	stem Carnivora
Sinopa rapax	stem Carnivora
Mesohippus bairdi	stem Equidae
Basilosaurus cetoides	stem Cetacea (1)
Artiocetus clavis	stem Cetacea (2, i.e. older than Basilosaurus)
Rodhocetus balochistanensis	stem cetacea (3, i.e. older than Artiocetus)
Archaeotherium mortoni	stem Whippomorpha
Icaronycteris index	Chiroptera (stem or crown)
Onychonycteris finneyi	Chiroptera (stem or crown)
Hyopsodus paulus	Laurasiatheria (stem or crown)
Apheliscus insidiosus	Laurasiatheria (stem or crown)
Phenacodus intermedius	Laurasiatheria (stem or crown)
Mesonyx obtusidens	Laurasiatheria (stem or crown)
Protungulatum donnae	Laurasiatheria (stem or crown)
Leptictis dakotensis	stem Eutheria (1)
Ukhaatherium nessovi	stem Eutheria (2)
Zalambdalestes lechei	stem Eutheria (2)
Maelestes gobiensis	stem Eutheria (2)
Eomaia scansoria	stem Eutheria (3)

Table S6: Fossil calibrations (My) used for the node-dating analysis. For each calibration, the calibrated node is referred to by two species, of which the node is the last common ancestor.

**Species 1**	**Species 2**	**Upper calibration**	**Lower calibration**
Cyclopes didactylus	Tamandua tetradactyla	61.1	15.97
Bradypus tridactylus	Choloepus sp.	40.6	15.97
Cyclopes didactylus	Choloepus sp.	65.5	31.5
Cyclopes didactylus	Dasypus novemcinctus	71.2	58.5
Dugong dugon	Trichechus manatus	40.6	31.4
Elephantulus rufescens	Rhynchocyon cirnei	56	15.97
Dugong dugon	Procavia capensis	71.2	55.6
Ochotona sp.	Oryctolagus cuniculus	61.1	48.4
Nycteris thebaica	Saccopteryx bilineata	58.9	40.2
Hipposideros commersoni	Rhinolophus creaghi	56	37.1
Megaderma lyra	Craseonycteris thonglongyai	48.8	33.8
Myotis lucifugus	Tadarida brasiliensis	56	37.1
Myotis lucifugus	Natalus stramineus	61.1	48.4
Pteronotus parnellii	Artibeus jamaicensis	40.6	28.3
Furipterus horrens	Noctilio sp.	34	5.332
Hipposideros commersoni	Myotis lucifugus	61.1	48.4
Canis lupus familiaris	Phoca vitulina	56	37.1
Ailuropoda melanoleuca	Phoca vitulina	48.8	33.8
Mephitis mephitis	Phoca vitulina	48.8	33.8
Procyon lotor	Meles meles	40.6	27.6
Procyon lotor	Mephitis mephitis	40.6	30.9
Felis silvestris	Prionodon linsang	40.6	28.3
Suricata suricatta	Canis lupus familiaris	65.8	37.1

Table S6 (continued): Fossil calibrations (My) used for the node-dating analysis. For each calibration, the calibrated node is referred to by two species, of which the node is the last common ancestor.

**Species 1**	**Species 2**	**Upper calibration**	**Lower calibration**
Nycticebus coucang	Otolemur garnettii	56	37.1
Tarsius syrichta	Homo sapiens	58.9	37.1
Homo sapiens	Saimiri sciureus	56	28.3
Lemur catta catta	Otolemur garnettii	56	37.1
Tupaia glis	Ptilocercus lowii	65.8	37.1
Equus caballus	Tapirus indicus	61.1	55.5
Erinaceus europaeus	Sorex araneus	84.2	61.1
Erinaceus europaeus	Podogymnura truei	48.8	28.3
Lama glama	Tragulus napu	65.8	52.5
Sus scrofa	Pecari tajacu	37.3	15.97
Hippopotamus amphibius	Tursiops truncatus	61.1	52.5
Tursiops truncatus	Caperea marginata	48.8	33.8
Ictidomys tridecemlineatus	Aplodontia rufa	58.9	45.7
Ictidomys tridecemlineatus	Gliridae	61.1	48.4
Dinomys branickii	Chinchilla lanigera	37.3	24.5
Cavia porcellus	Dasyprocta punctata	37.3	24.5
Anomalurus beecrofti	Pedetes sp.	56	37.1
Hystrix sp.	Cavia porcellus	56	33.8
Geomyidae	Dipodomys heermanni	40.6	31.4
Dipodomys heermanni	Castor canadensis	61.1	52.4
Spalax sp.	Jaculus jaculus	58.9	45
Laonastes aenigmamus	Ctenodactylus gundi	48.8	28.3
Ictidomys tridecemlineatus	Hystrix sp.	65.8	55.6
Pedetes sp.	Castor canadensis	65.8	52.4

Table S7: Fossil age constraints (My) used for the tip-dating analysis.

Species	Upper calibration	Lower calibration
Eomaia scansoria	124.4	122.2
Maelestes gobiensis	83.6	72.1
Metacheiromys marshi	50.3	46.2
Ukhaatherium nessovi	83.6	72.1
Zalambdalestes lechei	83.6	72.1
Leptictis dakotensis	37.2	33.3
Moeritherium lyonsi	37.2	33.9
Notharctus tenebrosus	50.3	46.2
Protungulatum donnae	66.0	63.3
Apheliscus insidiosus	55.8	50.3
Phenacodus intermedius	56.8	55.8
Protolipterna ellipsodontoides	59.0	57.5
Hyopsodus paulus	50.3	46.2
Didolodus multicuspis	54.0	51.0
Carodnia vieirai	59.0	57.5
Thomashuxleya externa	54.0	51.0

Table S7 (continued): Fossil age constraints (My) used for the tip-dating analysis.

**Species**	**Upper calibration**	**Lower calibration**
Vulpavus ovatus	50.3	46.2
Vulpavus profectus	50.4	46.2
Sinopa rapax	50.5	46.2
Dawsonolagus antiquus	54	53
Gomphos elkema	56	55
Rhombomylus turpanensis	54	53
Tribosphenomys minutus	59	57
Paramys delicatus	55.4	50.3
Cocomys lingchaensis	56	55
Mesohippus bairdi	38.0	33.9
Basilosaurus cetoides	40.4	37.2
Mesonyx obtusidens	50.3	46.2
Archaeotherium mortoni	38.0	33.9
Artiocetus clavis	48.4	46
Rodhocetus balochistanensis	48.4	46
Icaronycteris index	52.5	50.3
Onychonycteris finneyi	52.5	50.3

Supplementary Figures

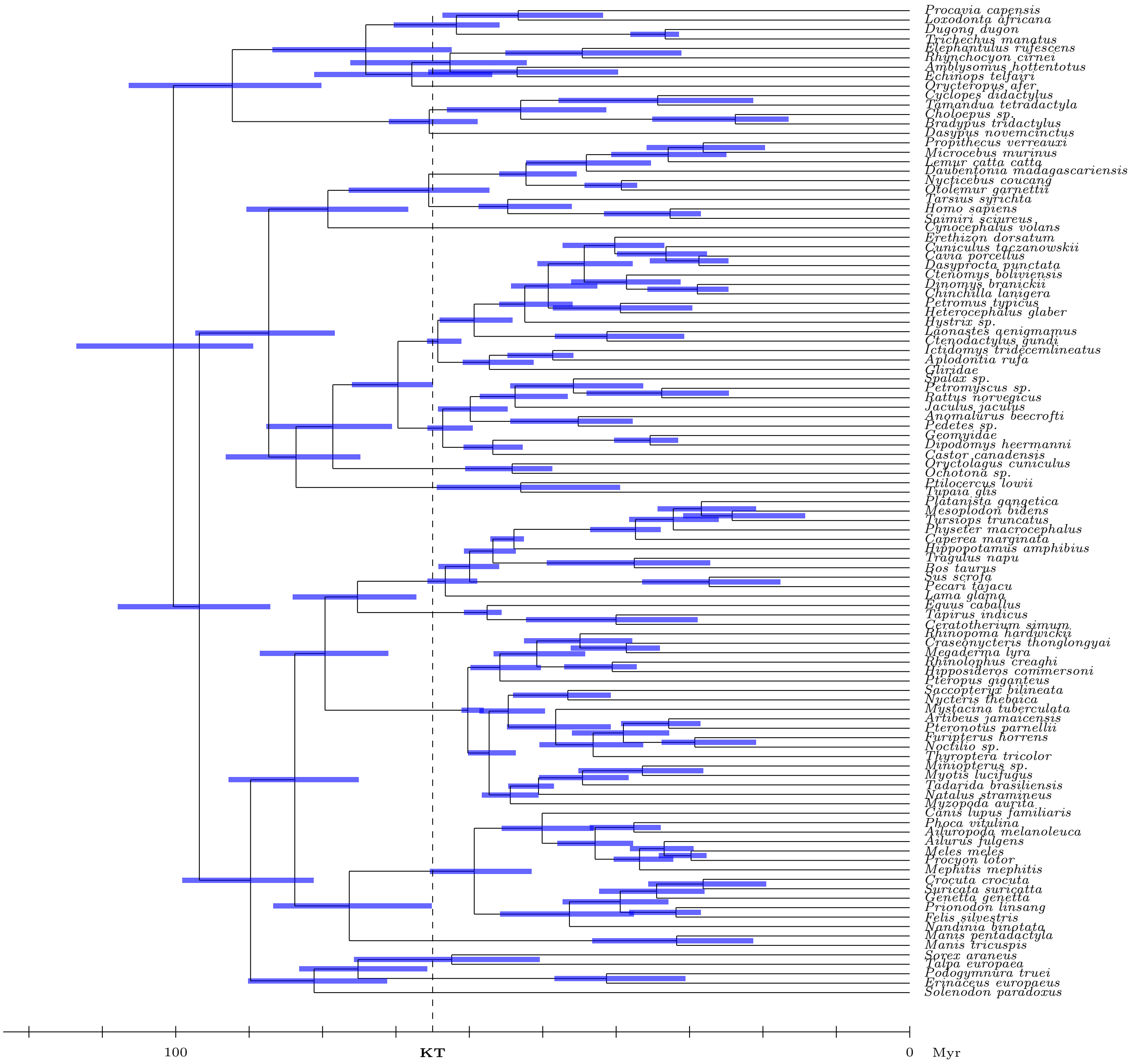

Figure S1. Inferred divergence times (posterior median, blue bars: 95% credible intervals) under the whitenoise clock, using the node-dating approach.

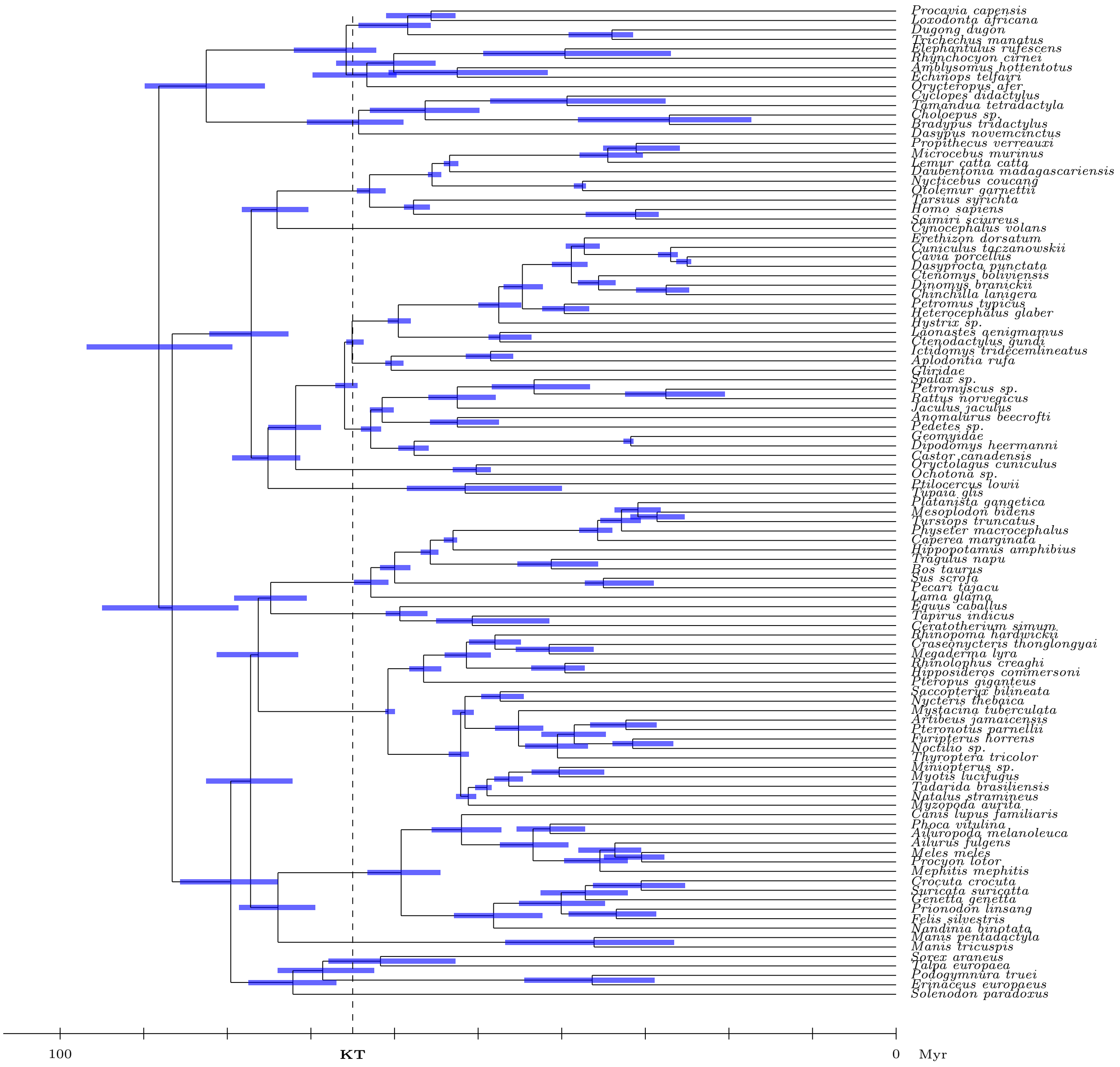

Figure S2. Inferred divergence times (posterior median, blue bars: 95% credible intervals) under the Brownian clock, using the node-dating approach.

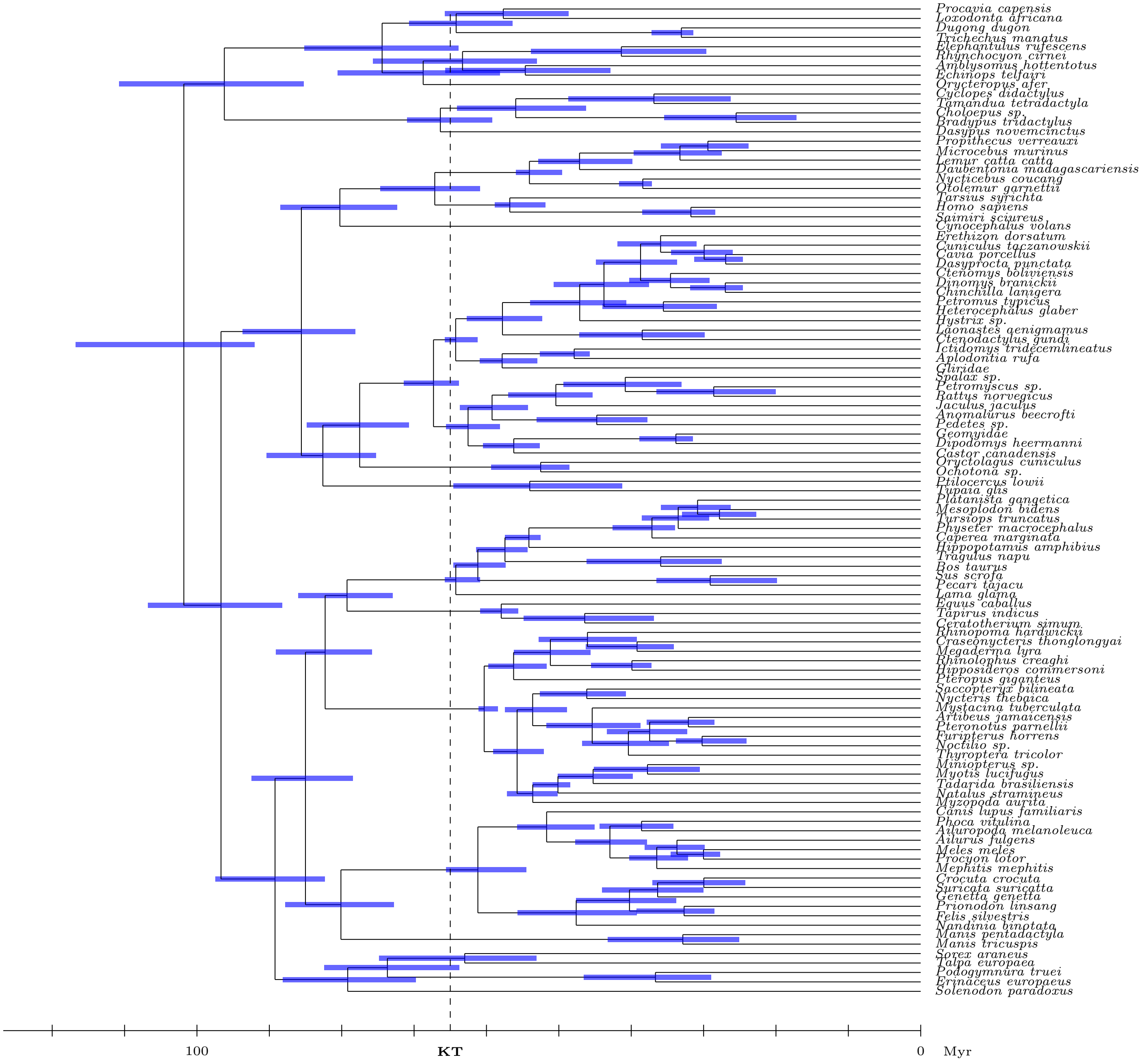

Figure S3. Inferred divergence times (posterior median, blue bars: 95% credible intervals) under the mixed clock, using the node-dating approach.

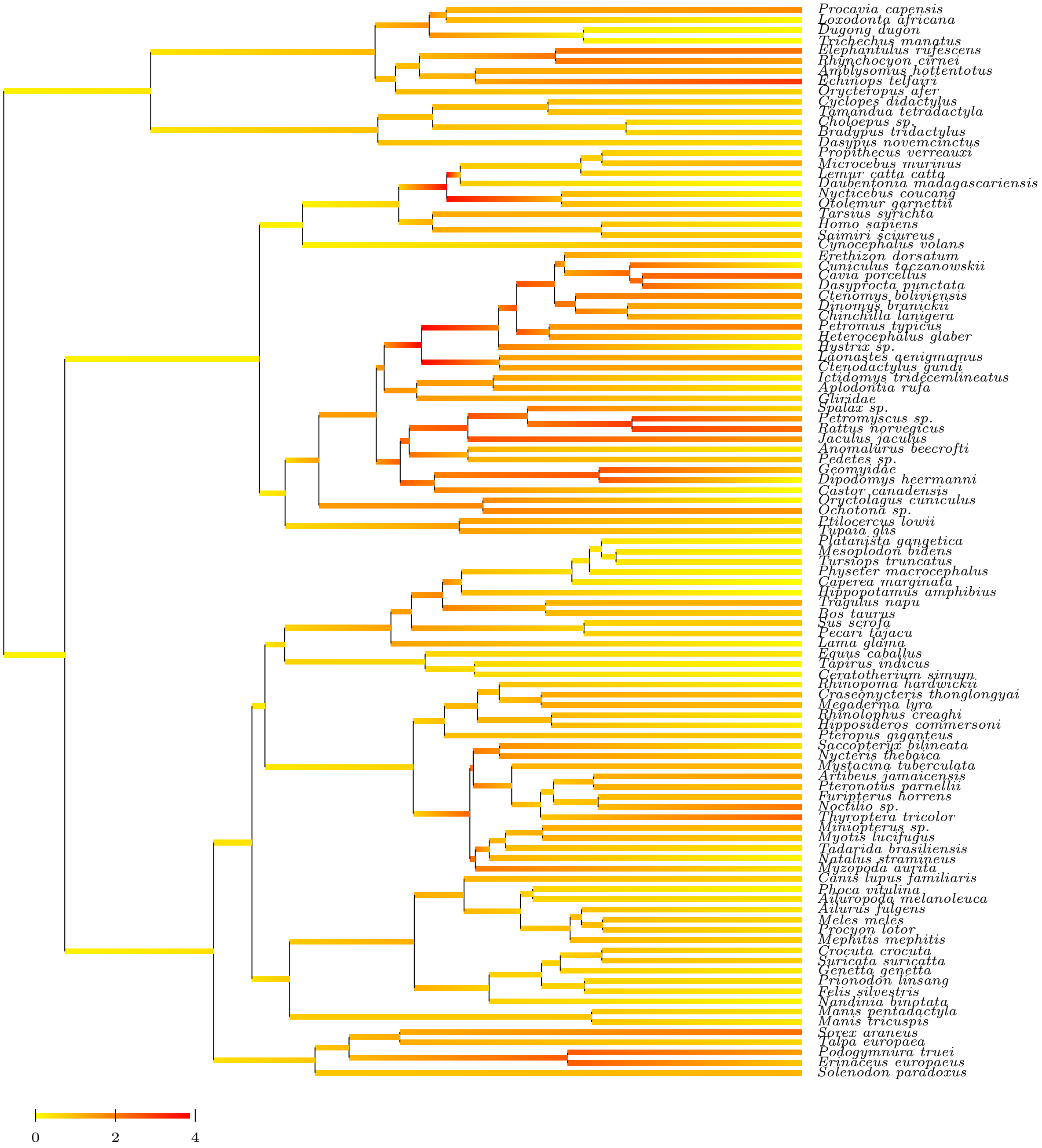

Figure S4. Inferred history of rate variation under the Brownian clock, using the node-dating approach.

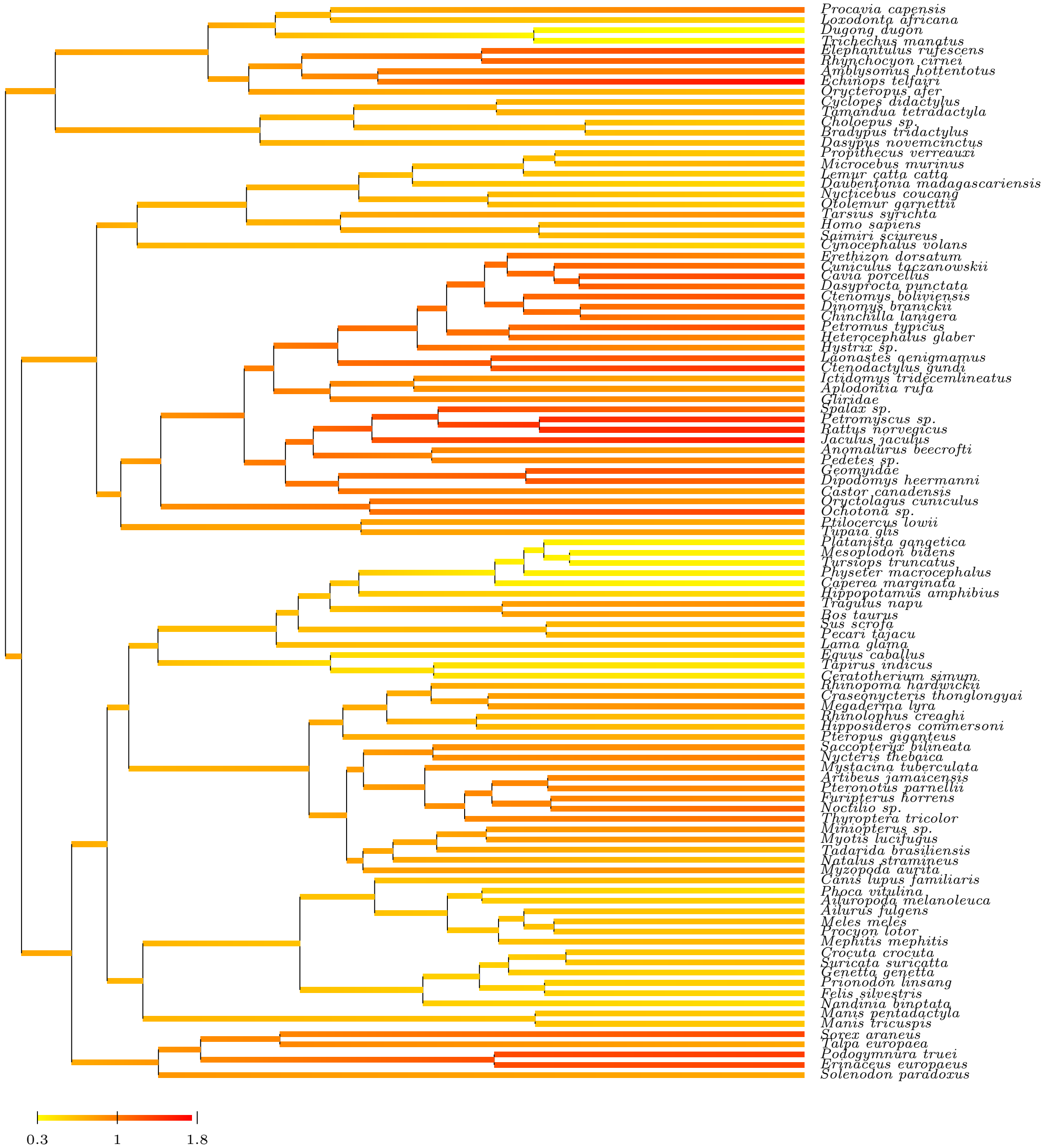

Figure S5. Inferred history of long-term rate variation (Brownian component of the mixed clock) using the node-dating approach.

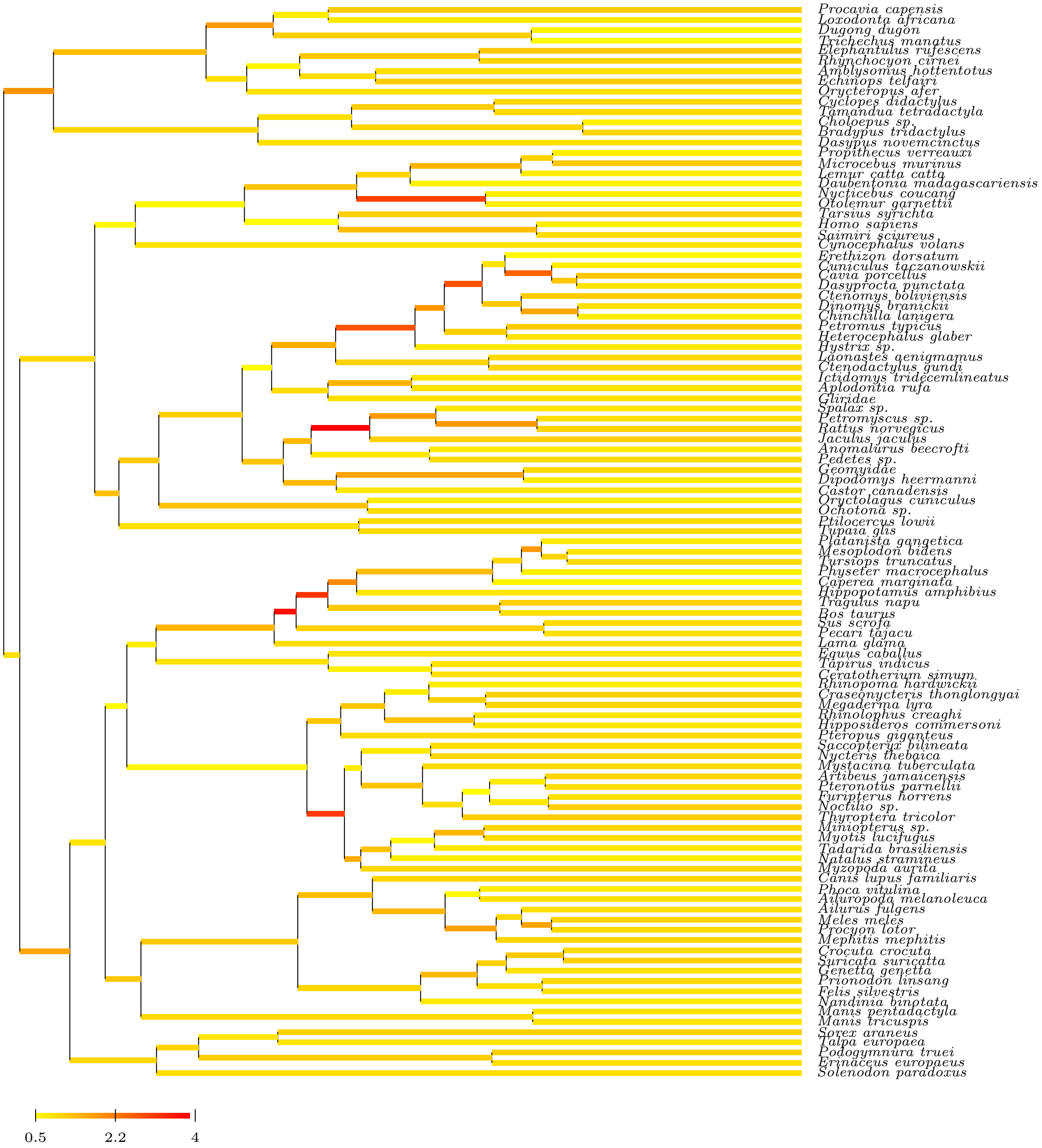

Figure S6. Inferred history of short-term rate variation (whitenoise component of the mixed clock) using the node-dating approach.

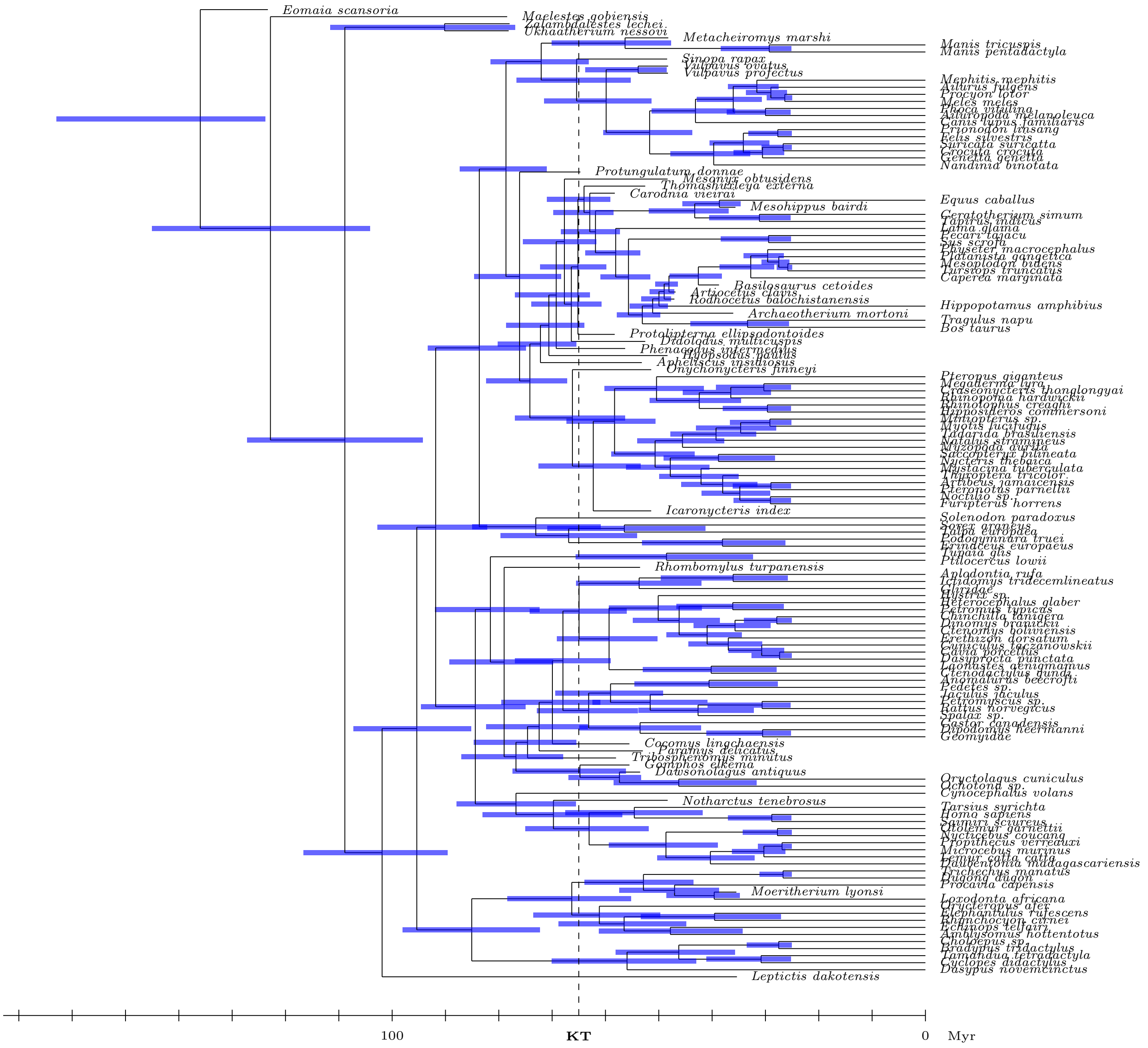

Figure S7. Inferred divergence times (posterior median, blue bars: 95% credible intervals) under the whitenoise clock, using the tip-dating approach.

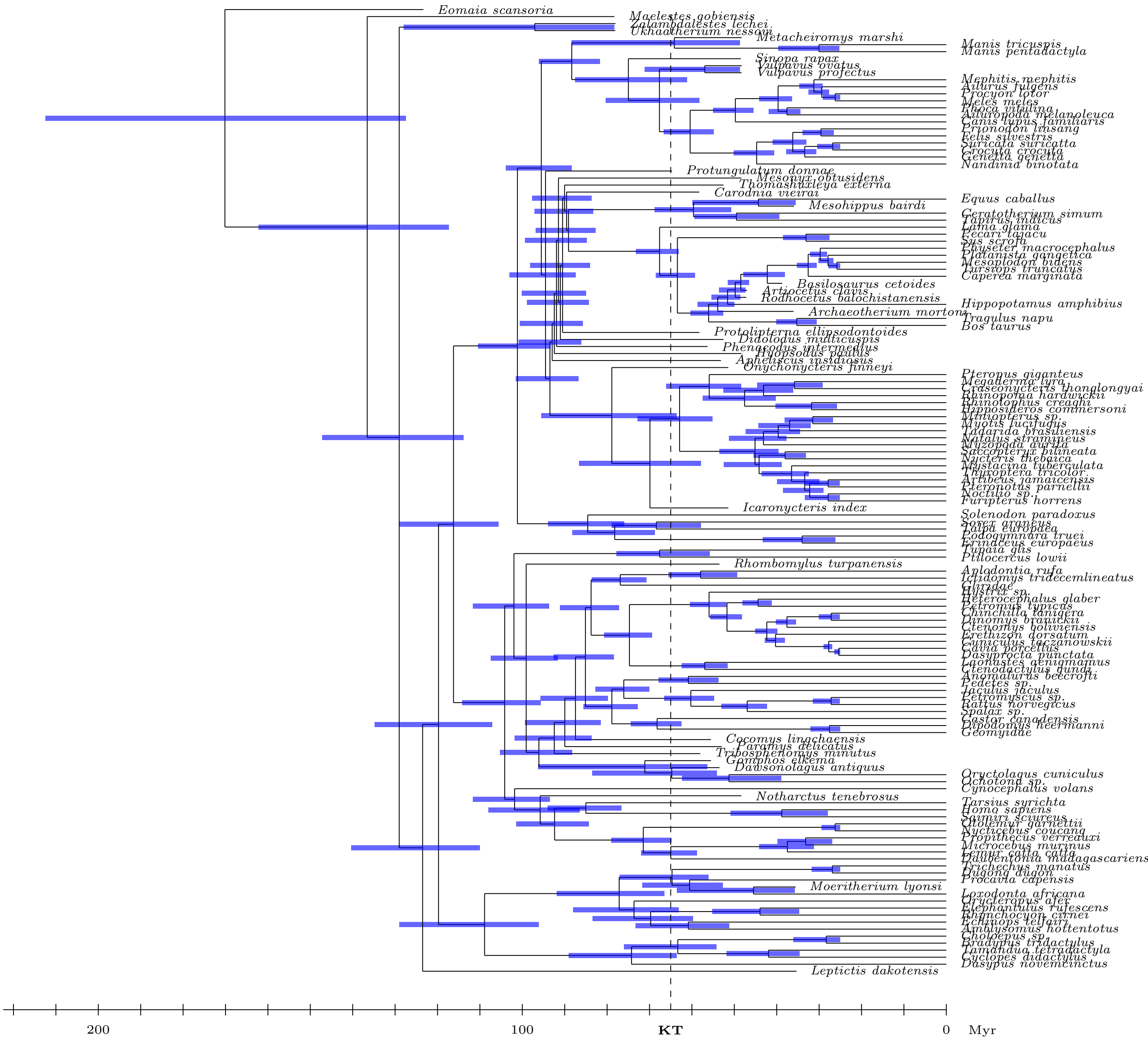

Figure S8. Inferred divergence times (posterior median, blue bars: 95% credible intervals) under the Brownian clock, using the tip-dating approach.

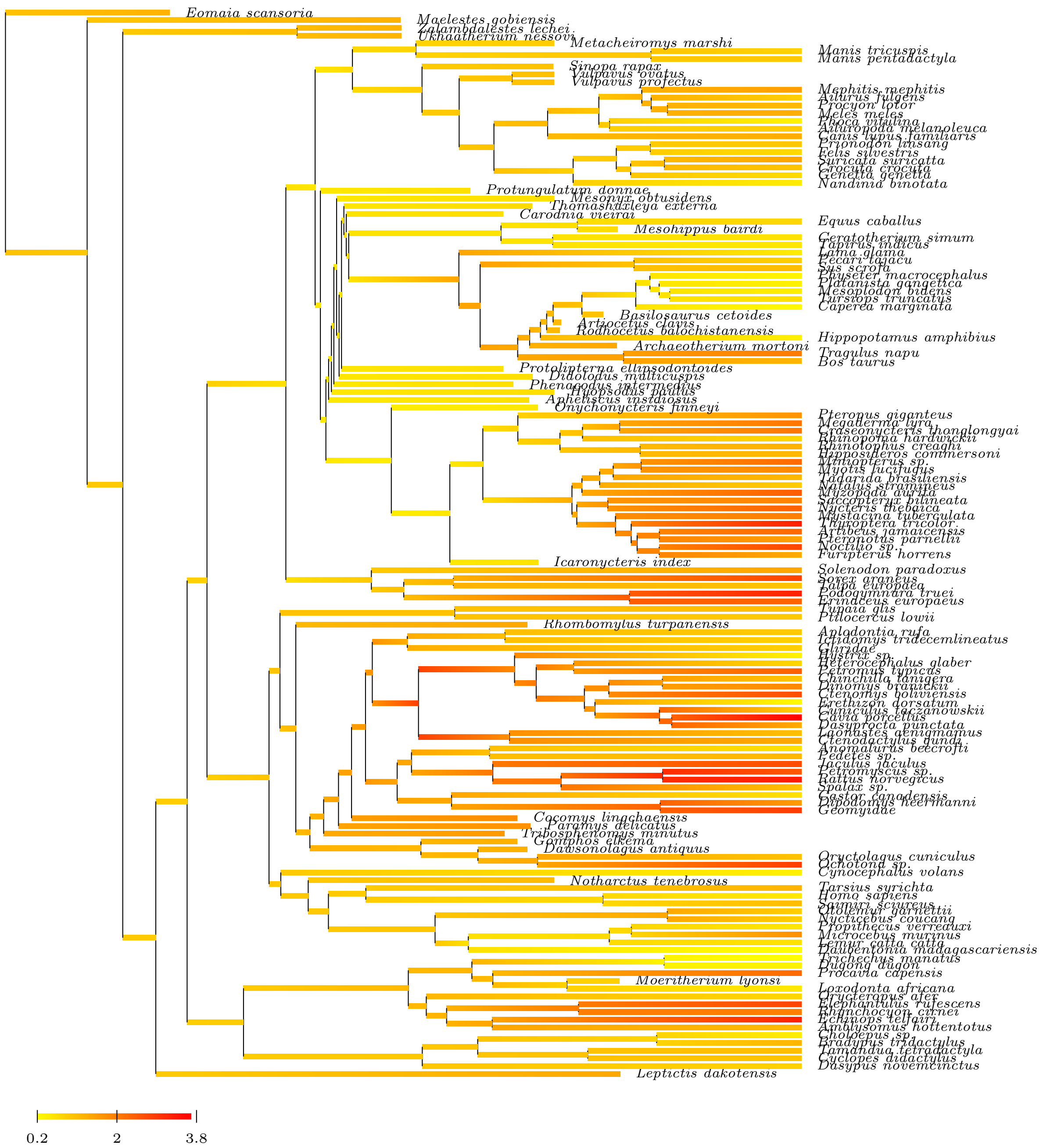

Figure S9. Inferred history of rate variation under the Brownian clock, using the tip-dating approach.

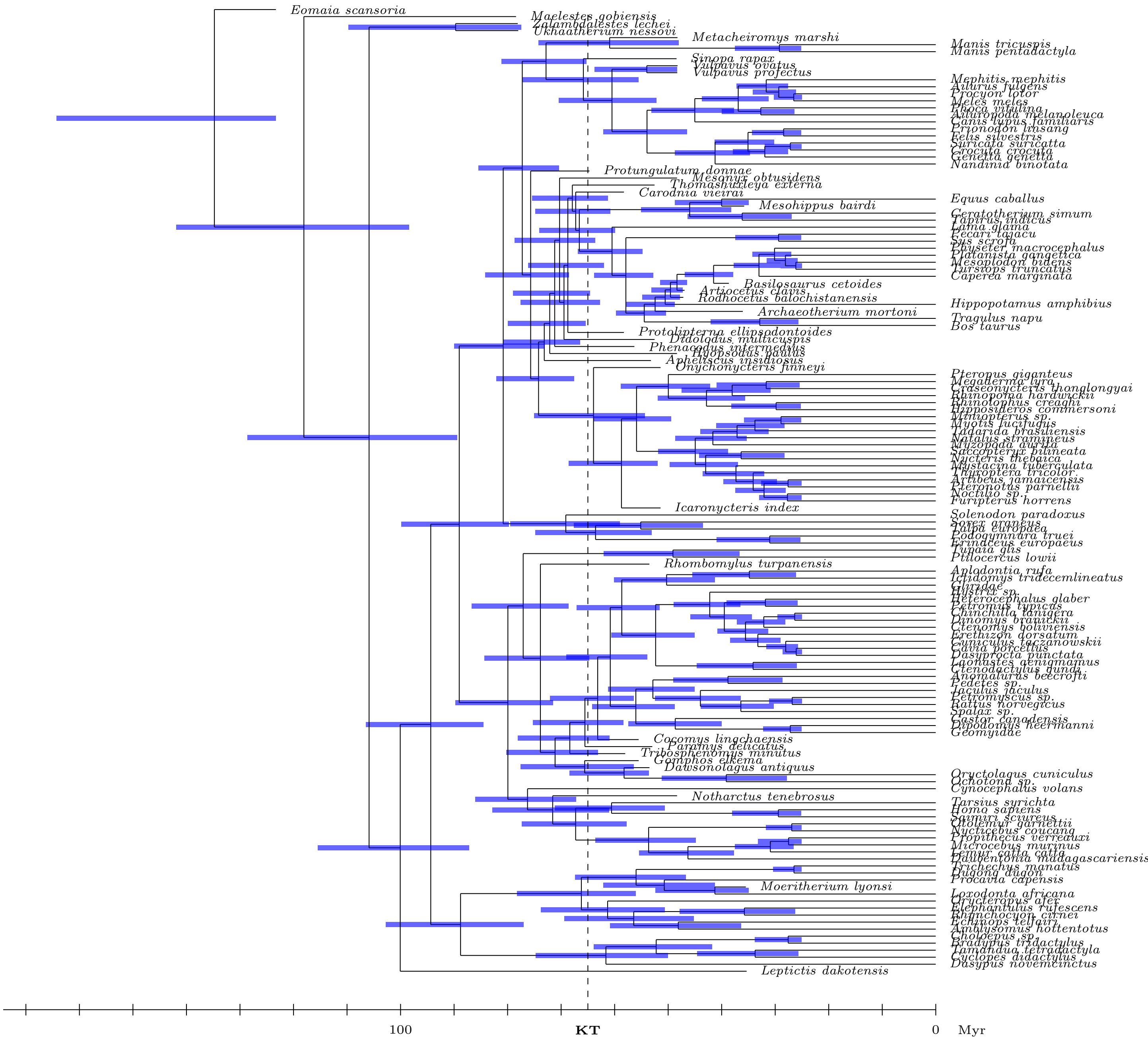

Figure S10. Inferred divergence times (posterior median, blue bars: 95% credible intervals) under the mixed clock, using the tip-dating approach and with morphological characters.
